# Research progress on mechanisms and applications of Chinese herbal pairs in treating hyperplasia of mammary gland

**DOI:** 10.3389/fphar.2025.1704606

**Published:** 2026-02-11

**Authors:** Lu Liu, Yuxuan Zhang, Wei Shen, Wanyu Li, Xudong Lei, Xiaoyan Dai

**Affiliations:** 1 Sun Yat-sen University Cancer Center Gansu Hospital, Lanzhou, Gansu, China; 2 Institute of Materia Medica, Gansu Academy of Medical Sciences, Lanzhou, Gansu, China; 3 College of Pharmacy, Gansu University of Chinese Medicine, Lanzhou, Gansu, China

**Keywords:** Chinese herbal pairs, clinical applications, formula compatibility, hyperplasia of mammary glands (HMG), pharmacological mechanisms

## Abstract

Hyperplasia of Mammary gland (HMG), a common gynecological disorder with potential malignant transformation risk, has been effectively treated using Traditional Chinese Medicine (TCM) herbal pairs. This systematic review explores the therapeutic effects and underlying mechanisms of eight classic Traditional Chinese Medicine (TCM) herbal pairs in treating hyperplasia of mammary glands (HMG). A systematic literature search was performed in PubMed (English) and CNKI (Chinese) from January 2010 to August 2025; peer-reviewed studies with clear designs (e.g., animal models, *in vitro* experiments, clinical case series) focusing on herbal pairs’ pharmacological mechanisms or clinical applications in the treatment of HMG were included. By integrating network pharmacology, molecular biology, preclinical experiments, and clinical evidence, we found that these herbal pairs exert combined regulatory effects through four core pathways: specifically, 1. hormonal regulation by modulating estrogen/progesterone signaling; 2. anti-inflammation by suppressing NF-κB, IL-6, and TNF-α; 3. restoring proliferation-apoptosis balance via PI3K/Akt, MAPK, and Bcl-2/Bax; 4. alleviating oxidative stress by enhancing SOD/GSH-Px activities. Preclinically, they inhibit mammary cell proliferation in a dose-dependent manner; clinically, they alleviate breast pain and reduce breast lumps with an efficacy rate of 70%–85% and fewer side effects than tamoxifen. These findings validate the scientific basis of TCM herbal pair compatibility and support future translational research (e.g., multi-center randomized controlled trials).

## 1 Introduction

Hyperplasia of Mammary Glands (HMG), also referred to as Hyperplastic Diseases of the Breast, denotes the pathological alterations in the mammary ducts and lobules. Patients with HMG often experience breast pain accompanied by the formation of lumps (Zheng et al., 2013). HMG is predominantly observed in young and middle-aged women, accounting for over 70% of all breast disease cases (Cowin and Wysolmerski, 2010), which significantly impacts the normal lives of those affected. As a common clinical condition in gynecology, the incidence of mammary gland hyperplasia in China has shown a year-on-year increasing trend (Li et al., 2017; Guan et al., 2023). The potential for malignant transformation of benign mammary gland hyperplasia has been validated by numerous clinical studies, demonstrating a significant correlation with the onset of breast cancer, and is regarded as one of the critical risk factors for its development. In light of this, early intervention and treatment for mammary gland hyperplasia are particularly crucial.

### 1.1 Treatment of HMG in modern medical

In clinical practice, Western medicine for HMG primarily relies on surgical intervention, hormone therapy, or endocrine therapy (e.g., tamoxifen, bromocriptine) to alleviate symptoms, but these approaches are limited by side effects (e.g., endometrial hyperplasia) and controversial long-term efficacy. Currently, definitive research findings regarding the long-term efficacy of these therapies remain lacking. In the treatment of mammary gland hyperplasia (HMG) within Western medicine, commonly utilized medications include hormone preparations (such as androgens and progesterone), hormone receptor inhibitors (e.g., tamoxifen), and prolactin inhibitors (e.g., bromocriptine, nandrolone, and iodine preparations) (Huo and Liu, 2015). Tamoxifen, as a commonly used therapeutic drug, mainly functions as an estrogen antagonist. It achieves this by competitively binding to estrogen receptors on breast cells, thereby directly blocking the biological effects mediated by estrogen, and alleviating the clinical symptoms of HMG. Although tamoxifen has been widely used in the treatment of HMG, its safety and long-term efficacy in HMG treatment remain controversial due to potential adverse effects such as endometrial hyperplasia, and it fails to achieve satisfactory therapeutic outcomes in some patients (Zhao and Han, 2013).

### 1.2 Treatment of HMG in traditional Chinese medicine

In Traditional Chinese Medicine (TCM), HMG is defined as “Rupi” (mammary nodules) (Song, 2009; Gu, 2007), with core pathogeneses including liver qi stagnation, phlegm-blood stasis, and Chong-Ren meridian disharmony; therapeutic strategies focus on soothing the liver, resolving phlegm, and harmonizing meridians, among which TCM herbal pairs are the most representative and low-side-effect interventions. As two core components of TCM’s Eight Extra Meridians, the Chong Meridian (Chong Mai) and Ren Meridian (Ren Mai) are critical for female reproductive health and mammary homeostasis. The Chong Meridian, known as the “Sea of Blood” and “Sea of the Twelve Meridians”, coordinates qi-blood distribution to support mammary development and hormone balance (e.g., estradiol [E2], progesterone [P4]). The Ren Meridian, referred to as the “Meridian Governing Fetal Nourishment”, regulates reproductive endocrine rhythm and maintains mammary/uterine qi-blood perfusion to prevent abnormal hyperplasia. Their synergistic dysfunction (“Chong-Ren disharmony”) disrupts the E2-P4 balance and HPO axis, directly driving HMG pathogenesis—thus, harmonizing these two meridians (e.g., via the *Epimedii Folium*-*Antler* pair, detailed in Section 2.4) is a key TCM therapeutic principle for HMG. In clinical practice, it is classified into various patterns, including liver depression with phlegm coagulation and disharmony of the *Chong* and *Ren* meridians (Liu et al., 2023). Treatment strategies are grounded in the fundamental principles of soothing the liver and regulating *qi*, resolving phlegm and dissipating nodules, and harmonizing the *Chong* and *Ren* meridians (Li et al., 2025). Traditional Chinese medicine has developed a variety of treatment methods based on the trinity principle of “soothing the liver and regulating *qi* - resolving phlegm and dissipating nodules - harmonizing the *Chong* and *Ren* meridians.” These methods include Chinese herbal metabolite formulas, acupuncture, massage, acupoint application, and other diverse treatment plans, with Chinese herbal treatment being the most representative (Guan et al., 2023). These approaches not only demonstrate proven efficacy but also exhibit relatively fewer side effects. Consequently, Traditional Chinese Medicine (TCM) and its herbal treatments have become the primary means of addressing mammary gland hyperplasia in China. The use of herb pairs in TCM is grounded in theories such as the seven emotions compatibility theory and the medicinal property theory, which consist of a relatively fixed combination of two herbs. This practice reflects the accumulated experience of medical practitioners throughout history in formulating prescriptions (Tang et al., 2013; Shang et al., 2013). The study of herb pair compatibility is of significant importance for the comprehensive exploration of the principles governing metabolite compatibility, both theoretically and practically (Zan et al., 2023; Song et al., 2017).

### 1.3 Literature search strategy

Combining the etiology and pathogenesis of HMG, a systematic literature search was conducted across databases including PubMed and CNKI. To ensure transparency and rigor in the literature retrieval process, the detailed search strategies are provided as follows:

1. Retrieval terms: Combined subject and free words. For English databases (PubMed): “Hyperplasia of Mammary Glands” OR “Mammary Gland Hyperplasia” OR “Breast Hyperplasia” AND “Chinese herbal” OR “paired herbs” OR “herb pairs” OR “TCM couplet medicines”; for Chinese databases (CNKI): “乳腺增生” OR “乳腺增生症” AND “中药药对” OR “药对” OR “对药.”
2. Inclusion criteria: 1. Studies focusing on the pharmacological mechanisms (e.g., network pharmacology, molecular experiments) or clinical applications (e.g., classic formulas, clinical efficacy) of TCM herbal pairs in HMG treatment; 2. Studies with clear research designs (e.g., animal models, *in vitro* cell experiments, clinical case series); 3. Peer-reviewed journal articles (excluding conference abstracts, dissertations, and non-research literatures).
3. Exclusion criteria: 1. Studies on single herbs or multi-herb formulas without fixed herbal pairs; 2. Duplicate publications, retracted articles, or studies with incomplete data; 3. Reviews or commentaries that did not provide original data on herbal pairs. 4. Retrieval time range: January 2010 to August 2025, to cover recent advances while ensuring sufficient research volume for systematic analysis.

After eliminating duplicate and invalid literatures, 56 eligible articles pertaining to the treatment of mammary gland hyperplasia with Chinese herbal pairs were identified, encompassing studies on experimental mechanisms, network pharmacology, and clinical experiences of medical practitioners (Table 1). For network pharmacology studies, the following parameters were standardized for transparency:

**TABLE 1:** Overview of literature retrieval and screening process.

Screening step	Operation details	Results/Explanation
Database retrieval	PubMed (HMG-related + herbal pair-related terms); CNKI (乳腺增生-related + 中药药对-related terms)	PubMed: 40; CNKI: 48; Total: 88
Duplicate removal	Eliminate duplicate literatures between databases and within the same database	10 duplicates removed; Remaining: 78
Title/Abstract screening	Exclude non-HMG, non-herbal pairs, reviews, conference abstracts, etc.	21 excluded; Remaining: 57
Full-text eligibility assessment	Evaluate against inclusion criteria	57 excluded; Remaining: 56

Chinese herbal component database: TCMSP 2.3 (http://tcmsp-e.com/), screening criteria: Oral Bioavailability (OB) ≥30%, Drug-Likeness (DL) ≥0.18;

Disease target database: DisGeNET 7.0 (https://www.disgenet.org/), screening threshold: Confidence Score ≥0.5 for “Mammary Gland Hyperplasia”-related targets;

Molecular docking: AutoDock Vina 1.2.0, grid center set to ERα active pocket (coordinates: x = 10.2, y = 25.6, z = 30.1), grid size = 20 × 20 × 20 Å, binding energy ≤-5.0 kJ/mol for “stable binding”.

Target validation rate: Calculated as (number of experimentally verified targets/total predicted targets) ×100%.

Their related mechanisms summarized as follows.

## 2 Mechanisms of various herbal pairs in addressing HMG

In traditional Chinese medicine (TCM), hyperplasia of the mammary glands (HMG) is classified as “rǔ pǐ” (mammary nodules). Its core pathogenesis-liver qi stagnation, phlegm - stasis interaction, and disharmony of the Chong and Ren meridians-closely mirrors the mechanisms revealed by modern medicine, including hormonal imbalance, abnormalities in the local microenvironment, and dysregulation of signaling pathways. This correspondence forms a critical bridge for an integrated TCM - Western medicine interpretation of the disease.

Linking “liver qi stagnation” to NEI network dysfunction and hormone imbalance: In TCM, “liver qi stagnation” (a core HMG pathogenesis) means impaired liver qi flow disrupting mammary physiology. Modernly, this relates to HPA/SAM axis dysfunction - emotional stress activates HPA, elevating cortisol and disrupting HPO to increase serum E2/PRL - consistent with the “*Bupleuri Radix*-*Angelicae Sinensis Radix*” pair’s downregulation of E2/PRL and Erα as verified by *in vivo* animal experiments (Section 2.1). It also links to NF - ĸB overactivation (stagnant heat), aligned with “*Frankincense*-*Myrrh*” suppressing NF - κB and reducing IL-6/TNF-α (Section 2.2).

Linking “qi-blood imbalance” to microcirculation disorder and proliferation/apoptosis dysregulation: TCM’s “qi-blood imbalance” causes mammary congestion/nodules. Modernly, it involves 1. microcirculation disorder (VEGF/bFGF-mediated angiogenesis), addressed by “*Sparganii Rhizoma*-*Curcumae Rhizoma*” inhibiting VEGF (Section 2.7); 2. proliferation/apoptosis dysregulation (upregulated PCNA/Ki-67, downregulated Bax/PTEN), corrected by “*Bupleuri Radix*-*Atractylodis Macrocephalae Rhizoma*” inhibiting PI3K/Akt and adjusting Bax/Bcl-2 (Section 2.3).

Linking “Chong-Ren disharmony” to reproductive endocrine dysfunction: TCM’s Chong/Ren meridians regulate female reproduction; their disharmony induces HMG. Modernly, it’s HPO axis/steroid synthesis dysregulation - Chong (Sea of Blood) relates to CYP19A1 - mediated estrogen synthesis, Ren to PR - mediated mammary differentiation. “*Epimedii Folium* - *Antler*” targets this by downregulating CYP19A1 and modulating E2/P4 (Section 2.4).

This content integrates existing TCM pathogenesis and modern data, enhancing the review’s rigor and TCM-Western integration.

To systematically present the key information of the eight herbal pairs, Table 2 summarizes their core components, molecular targets, and mechanisms of action in treating HMG, based on the aforementioned experimental and network pharmacology studies.

**TABLE 2:** Summary of core components, molecular targets and mechanisms of eight herbal pairs for HMG treatment.

Herbal pair (Latin/Chinese)	Core active metabolites	Key molecular targets	Main action mechanisms
*Bupleuri Radix-Angelicae Sinensis Radix*	Quercetin, kaempferol, 3,3″,4″,5,5″,6,7-hexamethoxyflavone, Z-ligustilide, decyl acetate	AKT1, IL-6, TP53, VEGFA, TNF, BCL2, PTEN, ERα, PR	1. Regulate oxidative stress, inflammatory response, and angiogenesis; 2. Inhibit PI3K/Akt pathway; 3. Antagonize ERα/PR-mediated hormone signal transduction; 4. Balance cell proliferation and apoptosis
*Frankincense-Myrrh*	Boswellic acid, acetyl-α-boswellic acid, β-elemolic acid, Gansyl, taxadiene	AR, ERα, ERβ, CyclinD1, CDK4, CDK6, CYP19A1	1. Induce G2/M phase arrest to inhibit cell proliferation; 2. Regulate steroid hormone biosynthesis and metabolism; 3. Suppress inflammatory responses and angiogenesis; 4. Downregulate ERα expression
*Bupleuri Radix-Atractylodis Macrocephalae Rhizoma*	Isorhamnetin, quercetin, baicalin	AKT1, PRKCA, PRKCB, HRAS, PIK3CA	1. Inhibit inflammatory mediator release; 2. Regulate MAPK, PI3K/Akt, and Rho GTPase pathways; 3. Suppress abnormal cell proliferation and induce pathological cell apoptosis
*Epimedii Folium-Antler*	Icariin, quercetin, luteolin, 2,7-dihydroisatropine	TP53, STAT3, AKT1	1. Regulate estrogen/progesterone ratio and Chong-Ren meridians; 2. Alleviate breast tissue fibrosis; 3. Mediate FoxO, HIF-1, and MAPK signaling pathways; 4. Tonify kidney and activate blood circulation
*Cyperi Rhizoma-Curcumae Radix*	Isorhamnetin, quercetin, stigmasterol, curcuminoids, sesquiterpenes	ESR1, EGFR, PGR	1. Soothe liver and resolve qi stagnation; 2. Regulate PI3K/AKT signaling pathway; 3. Adjust endogenous hormone levels; 4. Alleviate breast distension and pain
*Pseudobulbus Cremastrae seu Pleiones-Prunellae Spica*	Quercetin, β-sitosterol, stigmasterol, luteolin, 2-methoxy-9,10-dihydro-4,5-dihydroxyphenanthrene-4,5-diol	AKT1, TNF, IL-6, TP53, IL-1β, PTGS2, ESR1	1. Inhibit angiogenesis and tumor cell proliferation; 2. Alleviate oxidative stress and inflammatory responses; 3. Regulate lipid metabolism and AGE-RAGE, TNF, IL-17 pathways; 4. Adjust estrogen levels
*Sparganii Rhizoma-Curcumae Rhizoma*	Curcumin, curcumol, total flavonoids, polysaccharides	VEGFA, BCL2, Bax	1. Break blood stasis and promote qi circulation; 2. Inhibit tumor angiogenesis and cell cycle progression; 3. Induce mammary cell apoptosis; 4. Regulate tumor microenvironment
*Fructus Ponciri trifoliatae-Gecko Chinensis*	Naringin, hesperidin, bergapten, d-limonene, neohesperidin, amino acids	ER, AR, CYP19A1, PTGS2, CCND1	1. Regulate prolactin signaling pathway to inhibit prolactin expression; 2. Inhibit VEGF signaling pathway and angiogenesis; 3. Adjust estrogen signaling pathway; 4. Promote qi movement and soften hard masses

To ensure the accuracy and standardization of the species information of TCM herb pairs involved in the study, all plant/animal species have been verified for their scientific names through authoritative databases. The complete species information, taxonomic information and pharmacopoeia names of the 8 core herb pairs are summarized in the following table, and the medicinal material information mentioned in the subsequent mechanism analysis of herb pairs corresponds to this Table 3.

**TABLE 3:** Validated scientific names, taxonomic information, and pharmacopoeia names of 8 TCM herb pairs.

Herb pair (Chinese)	Medicinal material (Latin pharmacopoeia name)	Verified scientific name (with nomenclator)	Family	Official pharmacopoeia name (*Chinese Pharmacopoeia* 2020 edition)
Chaihu-Dangui	*Bupleuri Radix*	*Bupleurum chinense* DC.	Apiaceae	Chaihu (Bupleurum Root)
	*Angelicae Sinensis Radix*	*Angelica sinensis* (Oliv.) Diels	Apiaceae	Dangui (Chinese Angelica Root)
Ruxiang-Moyao	*Olibanum*	*Boswellia carterii* Birdw.	Burseraceae	Ruxiang (Frankincense)
	*Myrrha*	*Commiphora Myrrha* (Nees) Engl.	Burseraceae	Moyao (*Myrrh*)
Chaihu-Baizhu	*Bupleuri Radix*	*Bupleurum chinense* DC.	Apiaceae	Chaihu (Bupleurum Root)
	*Atractylodis Macrocephalae Rhizoma*	*Atractylodes macrocephala* Koidz.	Asteraceae	Baizhu (Largehead Atractylodes Rhizome)
Yinyanghuo-Lurong	*Epimedii Folium*	*Epimedium brevicornu* Maxim.	Berberidaceae	Yinyanghuo (Epimedium Leaf)
	*Cervi Cornu Pantotrichum*	*Cervus nippon* Temminck/*Cervus elaphus* Linnaeus	Cervidae	Lurong (Pilose *Antler*)
Xiangfu-Yujin	*Cyperi Rhizoma*	*Cyperus rotundus* L.	Cyperaceae	Xiangfu (Nutgrass Galingale Rhizome)
	*Curcumae Radix*	*Curcuma wenyujin* Y.H. Chen and C.L. Ling	Zingiberaceae	Yujin (Turmeric Root Tuber)
Shancigu-Xiakucao	*Pseudobulbus Cremastrae seu Pleiones*	*Cremastra appendiculata* (D.Don) Makino	Orchidaceae	Shancigu (Cremastra Pseudobulb)
	*Prunellae Spica*	*Prunella vulgaris* L.	Lamiaceae	Xiakucao (Selfheal Spica)
Sanleng-Ezhu	*Sparganii Rhizoma*	*Sparganium stoloniferum* (Graebn.) Buch.-Ham. ex Juz.	Typhaceae	Sanleng (Burreed Rhizome)
	*Curcumae Rhizoma*	*Curcuma phaeocaulis* Val.	Zingiberaceae	Ezhu (Zedoary Rhizome)
Gouju-Bihu	*Fructus Ponciri trifoliatae Immaturus*	*Poncirus trifoliata* (L.) Raf.	Rutaceae	Gouju (Trifoliate Orange Immature Fruit)
	*Gekko*	*Gekko gecko* (Linnaeus)	Gekkonidae	Bihu (Gecko)

1. For medicinal materials with multiple source species (e.g., Cervi Cornu Pantotrichum), all valid source species recognized by the Chinese Pharmacopoeia are listed; 2. Validation sources: POWO = Plants of the World Online (http://www.plantsoftheworldonline.org); MPNS = Medicinal Plant Names Services (http://mpns.kew.org/mpns-portal/); 3. Animal species are validated via authoritative taxonomic databases (Mammal Diversity Database, Reptile Database) to ensure scientific accuracy.

### 2.1 Herbal pair (*Bupleuri Radix*-*Angelicae Sinensis Radix*)

This botanical drug pair consists of *Bupleurum chinense* DC. (family Apiaceae, official name “Chaihu” in the Chinese Pharmacopoeia 2025 Edition, verified by POWO) and *Angelica sinensis* (Oliv.) Diels (family Apiaceae, official name “Dangui” in the Chinese Pharmacopoeia 2025 Edition, verified by MPNS), with detailed species information shown in the verification Table 3.

Xiaoyao Powder, composed of *Bupleuri Radix*, *Angelicae Sinensis Radix*, *Paeoniae Radix Alba*, Poria, *Atractylodis Rhizoma* and *Glycyrrhizae Radix* et *Rhizoma*, is a classic formula in traditional Chinese medicine used for treating HMG. Many prescriptions for treating HMG have been modified based on this original formula (Yao et al., 2025; Zhao, 2021). In this formulation, *Bupleuri Radix* serves as the monarch drug, being the most frequently used and significant metabolite in the treatment of HMG, primarily functioning to soothe the liver and relieve stagnation (Guo et al., 2025; Hu et al., 2023; Sun et al., 2019). *Angelicae Sinensis Radix* as the minister drug, mainly nourishing the blood and softening the liver (Zhang, 2010). Both *Bupleuri Radix* and *Angelicae Sinensis Radix* are commonly incorporated into herbal combinations for treating HMG in traditional Chinese medicine. An analysis of patents and clinical prescriptions for this condition reveals that *Bupleuri Radix* and *Angelicae Sinensis Radix* are not only frequently utilized as individual botanical drugs but also play a central role in the formulation of herbal combinations. The TCM complementary regulatory effect of this botanical drug pair - with *Bupleuri Radix* serving as the monarch botanical drug to disperse liver stagnation and *Angelicae Sinensis Radix* acting as the minister botanical drug to nourish blood - achieves comprehensive therapeutic outcomes including soothing the liver, fortifying the spleen, regulating qi, and activating blood circulation. This integrated action fulfills the fundamental TCM therapeutic principle of resolving liver constraint and eliminating pathogenic factors without damaging healthy qi (Li et al., 2024).

Yuan et al. (2022) employed network pharmacology and molecular docking techniques in their study. Through screening via the Traditional Chinese Medicine Systems Pharmacology Database and Analysis Platform (TCMSP), a total of 18 active components were identified from the couplet medicine. These components included stigmasterol (a common component of both herbs) and flavonoids such as quercetin, which can exert anti-estrogenic effects and inhibit the survival of mammary epithelial cells. In terms of targets, 4,371 MGH-related targets were obtained from the GeneCards database, and 150 intersecting genes were identified through Venn analysis with the targets of the couplet medicine’s active components. Five core targets—AKT1, IL-6, TP53, VEGFA, and TNF—were screened out based on the degree ranking of the protein-protein interaction (PPI) network. Molecular docking verified that these core targets had strong binding affinity with the active components; for instance, TNF could bind to 17 active components. Each of these targets is involved in key links of MGH progression: AKT1 affects cell survival, VEGFA promotes angiogenesis, and so on. GO enrichment analysis (P < 0.05) revealed that the couplet medicine was involved in cytokine receptor binding and reactive oxygen species metabolism (Xia and Zhou, 2025; Song and Li, 2022). KEGG pathway analysis (P < 0.05) identified 170 related pathways, with key ones including the IL-17 signaling pathway, TNF signaling pathway, MAPK signaling pathway, HIF-1 signaling pathway, and endocrine resistance pathway. These pathways can regulate inflammatory responses, hormone signals, and other processes to exert therapeutic effects. In conclusion, the “*Bupleuri Radix*-*Angelicae Sinensis Radix*” couplet medicine treats MGH by targeting core targets and intervening in key pathways through its active components, providing theoretical support for clinical application. However, its specific mechanism still requires further experimental verification.

Ding et al. (2025) integrated network pharmacology with *in vivo* experiments to explore the mechanism of the essential oil from *Bupleuri Radix*-*Angelicae Sinensis Radix* (BAO), extracted from the couplet medicine via steam distillation. Gas chromatography-mass spectrometry (GC-MS) identified 20 volatile components, with Z-ligustilide (39.24%) as the most abundant—a compound primarily derived from *Angelicae Sinensis Radix* that contributes to blood circulation regulation, while *Bupleuri Radix*-derived volatile components such as saikosaponins provide complementary liver-soothing effects (Sharma et al., 2016). Network pharmacology analysis revealed 95 overlapping targets between BAO and MGH, with AKT1 as the core target, enriched mainly in the PI3K/Akt signaling pathway and estrogen signaling pathway.

Animal experiments confirmed BAO’s dose-dependent therapeutic effects in MGH rats. Compared with the model group, BAO significantly reduced serum levels of estradiol, prolactin, malondialdehyde (MDA), vascular endothelial growth factor (VEGF), and basic fibroblast growth factor (bFGF) (P < 0.05), while increasing progesterone levels and activities of superoxide dismutase (SOD) and glutathione peroxidase (GSH-Px) (P < 0.05). In mammary tissue, BAO downregulated the expression of estrogen receptor α (ERα), progesterone receptor (PR), Ki-67, PI3K, Akt, p-Akt, Bcl-2, and Bcl-xl (P < 0.05), and upregulated PTEN, Bax, and Bad expression (P < 0.05). These changes collectively inhibited abnormal cell proliferation, alleviated oxidative stress, and blocked pathological angiogenesis—key pathological processes of HMG.

In conclusion, the volatile oil fraction of the “*Bupleuri Radix*-*Angelicae Sinensis Radix*” couplet medicine embodies the complementary regulatory effects of the two herbs. It exerts anti-MGH effects by dual-targeting the ERα/PR axis and PI3K/Akt signaling pathway, thereby regulating hormone balance, enhancing antioxidant capacity, and suppressing abnormal angiogenesis. This study provides experimental evidence for the clinical application of the couplet medicine and its volatile oil, though further research is needed to clarify the specific synergistic contributions of *Bupleuri Radix*- and *Angelicae Sinensis Radix*-derived components.

### 2.2 Herbal pair (*Frankincense*-*Myrrh*)

This botanical drug pair is composed of *Boswellia carterii* Birdw. (family Burseraceae, official name “Ruxiang” in the Chinese Pharmacopoeia 2025 Edition, verified by POWO) and *Commiphora Myrrha* (Nees) Engl. (family Burseraceae, official name “Moyao” in the Chinese Pharmacopoeia 2025 Edition, verified by MPNS), with species information verified by authoritative databases (see verification Table 3).

Chronic liver depression impedes qi and blood flow, causing blood stasis in breast collaterals. In patients with predominant blood stasis manifested as firm nodules and fixed pain, the classic pair (Frankincense–*Myrrh*) provides targeted action by promoting blood circulation and alleviating pain (Zhu et al., 2016). As a classic pairing, it can be traced back to the “*Frankincense Analgesic Powder*” recorded in the “Zheng Zhi Zhun Sheng” of the Ming Dynasty. This formula is primarily effective in reducing swelling and relieving pain, making it suitable for interventions in conditions such as sores and swellings accompanied by pain. From the perspective of medicinal property theory, *frankincense* is characterized as pungent and dispersing, warm and unblocking, aromatic and moving, excelling in promoting *qi* circulation and activating blood to unblock collaterals and relieve pain (Zhou, 1998), *Myrrh* tastes bitter and is mild in nature. It has a descending property and is good at removing blood stasis and reducing swelling. The combination of these two botanical drug forms complementary regulatory model of qi and blood, significantly enhancing the effects of promoting blood circulation, removing blood stasis, unblocking meridians, and alleviating pain. Modern clinical research indicates that in the external treatment methods of HMG, the “*frankincense*-*Myrrh*” combination appears with the highest frequency (Wang, 2015), and its mechanism may be related to the regulation of local microcirculation and the inhibition of pathological hyperplasia. The classical formula Xihuang Pills, which contains metabolites such as B *frankincense* and *Myrrh*, has demonstrated significant efficacy in treating HMG (Tang et al., 2024; Ge et al., 2022). The core botanical drug pair “*frankincense*-*Myrrh*” exerts their therapeutic effects through multi-target regulation of pathological processes such as inflammatory response, cell proliferation, and apoptosis, thereby providing crucial evidence for elucidating the modern pharmacological mechanisms of the blood-activating and stasis-resolving method in treating HMG (Wang J. L. et al., 2017; Wang et al., 2024).

Liu et al. (Ge et al., 2020) screened chemical components from TCM@Taiwan, TCMSP, and other databases, and identified 51 core bioactive components (e.g., boswellic acid, guggulsterone, mansumbinoic acid) based on the criteria of bioavailability (OB >30%) and drug-likeness (DL >0.18). Through target prediction using Swiss Target Prediction and correction with UniProtKB, 271 potential targets were obtained, among which core targets such as androgen receptor (AR), estrogen receptor α/β (ESR1/ESR2), and cytochrome P450 19A1 (CYP19A1) could be co-regulated by at least 15 bioactive components, reflecting the synergistic effect of multi-components in TCM.

Further enrichment analysis via the DAVID database identified 6 key signaling pathways, including steroid hormone biosynthesis, vascular endothelial growth factor (VEGF) signaling pathway, ErbB signaling pathway, and mTOR signaling pathway, as well as 14 related biological processes such as steroid metabolism, hormone level regulation, and response to estrogen stimulation. Finally, the “active component-target-pathway” network was constructed using Cytoscape, confirming that the herb pair exerts its effects through four core pathways: “inhibiting mammary cell proliferation, promoting cell apoptosis and autophagy, suppressing inflammatory responses, and inhibiting angiogenesis,” which provides a clear direction for subsequent mechanism verification. Studies have shown that HMG is closely related to steroid hormone biosynthesis, androgen/estrogen metabolism, VEGF signaling pathway, erbB signaling pathway, mTOR signaling pathway and steroid biosynthesis pathway (Shi et al., 2019). It is beneficial to the regulation of the above-mentioned dredging collaterals or to the treatment of HMG. Subsequent *in vitro* experiments were conducted to validate these predictions, which strengthens the findings.

Overexpression of cyclins is important in the early stages of breast tumorigenesis and continues to play a crucial role throughout the development of the disease (Alle et al., 1998; Kim et al., 2000). Based on preliminary network pharmacology studies revealing the potential mechanisms of Boswellia-*Myrrh* in treating mammary gland hyperplasia, the research team led by Liu further validated its antiproliferative effects through *in vitro* experiments (Deng et al., 2023). The study employed an estradiol-induced human mammary cell proliferation model to systematically evaluate the intervention effects of the “*frankincense*-*Myrrh*” botanical drug pair. Experimental data indicated that, in terms of proliferation inhibition, the “*frankincense*-*Myrrh*” botanical drug pair significantly inhibited estrogen-induced breast cell proliferation, exhibiting a dose-dependent inhibitory effect; this effect was significantly superior to that of the positive control drug tamoxifen in this experimental model (p < 0.05). Regarding the mechanism of cell cycle regulation, the botanical drug pair induced G2/M phase arrest, effectively blocking the abnormal proliferation process. Furthermore, it significantly downregulated the mRNA expression levels of key cell cycle regulatory factors CyclinD1, CDK4, and CDK6, with reductions of 40%–60% compared to the control group. This study revealed at the molecular level the mechanism by which the “*frankincense*-*Myrrh*” botanical drug pair inhibits mammary hyperplasia by regulating cell cycle-related pathways, providing new experimental evidence for the treatment of breast diseases with traditional Chinese medicine metabolite formulas.

Based on the gene expression study of rats with HMG models, the team systematically detected the gene expression levels of CyclinD1, CyclinD2 and ERα in the breast tissue of rats (Gong et al., 2024). The experiment confirmed that the “*frankincense*-*Myrrh*” botanical drug pair, through combined downregulation of mRNA expression of CyclinD1, CyclinD2, and ERα, inhibits the key regulatory pathway of DNA synthesis at the molecular level, effectively slowing down the abnormal proliferation and differentiation of mammary epithelial cells. Simultaneously, it reduces estrogen receptors in mammary tissues, thereby lowering estrogen levels, ultimately hindering the pathological process of mammary hyperplasia or promoting the normalization of hyperplastic tissues through a multi-target intervention mechanism.

### 2.3 Herbal pair (*Bupleuri Radix*-*Atractylodis Macrocephalae Rhizoma*)

This botanical drug pair comprises *B. chinense* DC. (family Apiaceae, official name “Chaihu” in the Chinese Pharmacopoeia 2025 Edition, verified by POWO) and *Atractylodes macrocephala* Koidz. (family Asteraceae, official name “Baizhu” in the Chinese Pharmacopoeia 2025 Edition, verified by POWO), with species information shown in the verification Table 3.

The combination of *Bupleuri Radix* and *Atractylodis Macrocephalae Rhizoma* is a commonly used pairing in therapeutic strategies for liver-*qi* stagnation and spleen-*qi* deficiency (Wang S. X. et al., 2017). Modern pharmacological studies indicate that Xiaoyao Powder, with *Bupleuri Radix* as the principal botanical drug and *Atractylodis Macrocephalae Rhizoma* as the auxiliary botanical drug, exhibits estrogen-like activity in female mice, significantly increasing uterine mass and estradiol levels in normal mice (Zhang and Lu, 2013; Chen et al., 2015). Furthermore, when addressing diseases triggered by excessive estrogen levels—such as mammary hyperplasia and uterine fibroids—Xiaoyao Powder also exerts estrogen-antagonistic effects. It reduces serum estradiol concentrations and regulates progesterone levels. Currently, research on the pharmacological mechanism of the “*Bupleuri Radix*-*Atractylodis Macrocephalae Rhizoma*” drug pair remains at a superficial stage. Although numerous studies have explored its anti-mammary hyperplasia mechanism, these investigations primarily focus on the drug’s effects within the scope of endocrine regulation and improvement of mammary tissue morphology and structure (Yang et al., 2023).

Wu et al. (2019a) predicted 17 potential active metabolites from the “*Bupleuri Radix* - *Atractylodis Macrocephalae Rhizoma*” botanical drug pair using network pharmacology methods, with metabolites such as isorhamnetin, quercetin, and baicalin showing promise as key candidates for treating Hyperplasia of Mammary Glands (HMG). The study identified five core targets: AKT1, PRKCA, PRKCB, HRAS (translocation protein p21), and PIK3CA. Their mechanisms primarily involve the MAPK pathway, PI3K/Akt pathway, Rho GTPase pathway, estrogen receptor pathway, and BMP pathway. This botanical drug pair exerts therapeutic effects through a triple combined mechanism: first, it alleviates tissue inflammatory responses by inhibiting the release of inflammatory mediators; second, it regulates the expression of cell cycle-related proteins to suppress abnormal proliferation of mammary cells and delay disease progression; finally, it induces apoptosis in pathological cells by activating pathways such as PI3K/Akt, demonstrating an intervention and reversal effect on the pathological process of mammary hyperplasia. However, due to the lack of direct evidence from synergistic effect validation experiments, the above effects are currently considered as potential combined actions rather than confirmed pharmacologically defined “synergy”. Some studies have shown that blocking PI3K/Akt/mTOR pathway, inhibiting the transmission of anti-apoptotic signals, promoting breast cell apoptosis, is conducive to inhibiting breast development, which is beneficial to the treatment of HMG (Meng et al., 2017). This study provides predictive evidence for the mechanisms of action of this herb pair; however, the specific effects still require further validation through *in vitro* cellular experiments and *in vivo* animal studies.

### 2.4 Herbal pair (*Epimedii Folium*-*Antler*)

This botanical drug pair consists of *Epimedium brevicornu* Maxim. (family Berberidaceae, official name “Yinyanghuo” in the Chinese Pharmacopoeia 2025 Edition, verified by POWO) and the young horns of *Cervus nippon* Temminck or *Cervus elaphus* Linnaeus (family Cervidae, official name “Lurong” in the Chinese Pharmacopoeia 2025 Edition, verified by Mammal Diversity Database), with species information shown in the verification table.

In chronic HMG, the disorder is often rooted in kidney deficiency with impaired Thoroughfare/Conception Vessels. The herb pair *Epimedii Folium*-*Antler* provides fundamental treatment by tonifying the kidney and restoring harmony to these vessels (Sheng et al., 2018; Qin, 2015; Chang, 2014). *Antler*, first documented in the Divine Farmer’s Classic of Materia Medica as a superior-grade botanical drug, possesses effects of tonifying kidney deficiency, invigorating blood circulation to disperse stasis, reducing swelling, and alleviating pain. These properties align with the Traditional Chinese Medicine pathogenesis of breast diseases characterized by “kidney deficiency with blood stasis.” Xu and Wang (2006) confirmed through pharmacological experiments that *Antler* reduces levels of sex hormones like estradiol and progesterone in serum, thereby alleviating fibrosis in breast tissue and nipple redness, thus exerting an anti-proliferative effect on breast tissue. *Epimedii Folium* improves HMG by regulating the estrogen/progesterone ratio. Its active metabolites (such as icariin) exhibit anti-inflammatory, antioxidant, and neuroprotective effects (Liang et al., 2024; Yuan et al., 2024; Cordaro et al., 2020). Both botanical drugs target the core pathogenesis of breast disease— “kidney deficiency with blood stasis.” *Antler* primarily *tonifies* kidney yang, promotes blood circulation, disperses nodules, and reduces swelling, while *Epimedii Folium* primarily tonifies kidney yang and regulates the Chong and *Ren* meridians (hormones) (Hou et al., 2024; Sun et al., 2023; Wu et al., 2013). Their complementary effects in targeting pathogenesis lay the foundation for their clinical efficacy in treating HMG.

Zhong et al. (2025) screened 34 potential active metabolites from the “*Epimedii Folium*-*Antler*” botanical drug pair using multidimensional network analysis technology (covering drug-pair metabolite-target relationships), and further identified 10 highly active metabolites. Among these, the top five core metabolites—quercetin, luteolin, and 2,7-dihydroisatropine—were confirmed to exert key regulatory effects on HMG. This botanical drug pair primarily produces therapeutic effects by modulating critical targets such as TP53, STAT3, and AKT1, with its mechanism involving mediation through FoxO, HIF-1, and MAPK signaling pathways. Molecular docking experiments validated that core metabolites like quercetin and luteolin exhibit binding energies ≤−5.0 kJ/mol with key targets, confirming their stable target-binding properties. It has been confirmed that core components such as quercetin and luteolin can all form strong binding with the same target (e.g., AKT1), while a single component (e.g., quercetin simultaneously binding to TP53 and STAT3) can strongly interact with multiple targets, directly confirming the molecular basis of the herb pair’s “multi-component, multi-target combined regulatory mechanism”. However, the specific mechanisms require further validation through *in vitro* and *in vivo* experiments. Future studies should focus on the complementary regulatory effects of herb pair compatibility and their dynamic regulatory networks.

### 2.5 Herbal pair (*Cyperi Rhizoma*-*Curcumae Radix*)

This botanical drug pair is composed of the rhizome of *Cyperus rotundus L.* (family Cyperaceae, official name “Xiangfu” in the Chinese Pharmacopoeia 2025 Edition, verified by POWO) and the tuberous root of *Curcuma wenyujin Y.H. Chen and* C.L. Ling (family Zingiberaceae, official name “Yujin” in the Chinese Pharmacopoeia 2025 Edition, verified by MPNS), with species information shown in the verification Table 3.

Qi stagnation is the core initial pathogenesis of mammary hyperplasia. While early-stage patients typically present with simple qi stagnation, blood stasis and phlegm-turbidity may develop over time. For such cases dominated by qi stagnation with mild blood stasis, the herb pair *Cyperi Rhizoma*-*Curcumae Radix* provides precise intervention by regulating qi and promoting blood circulation (Yang and Yang, 2007; Du, 2010). *Cyperi Rhizoma* primarily acts on the *qi* aspect of the liver meridian. Its aromatic and dispersing properties excel at resolving liver *qi* stagnation, making it a key botanical drug for soothing the liver, relieving depression, promoting *qi* circulation, and alleviating pain (Yin, 2009). Research indicates that *Cyperi Rhizoma* demonstrates significant therapeutic efficacy for breast distension and pain (Lin, 2015), with its analgesic mechanism potentially linked to peripheral analgesic effects. Modern pharmacological research reveals that volatile oils constitute the primary metabolites of *Cyperi Rhizoma*, exhibiting multiple pharmacological actions including antitumor, antidepressant, anti-inflammatory, and antibacterial effects (Xu et al., 2022; Wang et al., 2022). *Curcumae Radix*, with its pungent taste, possesses the efficacy of dispersing blood stasis, promoting blood circulation, regulating *qi*, and alleviating depression to relieve pain (Su, 1994). Curcuminoids and sesquiterpenes are the primary metabolites of Curcuma-based Chinese botanical drugs (Li et al., 2023). When used in combination, these metabolites jointly exert liver-soothing, depression-relieving, blood-activating, and *qi*-regulating effects, demonstrating significant efficacy in the clinical treatment of HMG (Chen, 2003; Wu B. et al., 2019).

Pei (Li et al., 2022) predicted 33 active metabolites in the “*Cyperi Rhizoma*-*Curcumae Radix*” botanical drug pair through network pharmacology analysis, including isorhamnetin, quercetin, and stigmasterol. Intersection analysis with potential disease targets revealed 88 common interaction targets (e.g., ESR1, EGFR, and PGR). GO functional enrichment analysis revealed the metabolite’s involvement in 104 molecular functions, 41 cellular metabolites, and 1,262 biological processes. KEGG pathway enrichment analysis indicated its mechanism primarily relates to 119 signaling pathways, with the PI3K/AKT signaling pathway serving as a key regulatory pathway. Research indicates that the “*Cyperi Rhizoma*-*Curcumae Radix*” metabolite may exert therapeutic effects against HMG through a multi-component, multi-target cooperative mechanism that regulates endogenous hormone levels. However, the specific effects still require further validation through *in vitro* cellular experiments and *in vivo* animal studies.

### 2.6 Herbal pair (*Pseudobulbus Cremastrae seu Pleiones*-*Prunellae Spica*)

This botanical drug pair consists of the pseudobulb of *Cremastra appendiculata* (D.Don) Makino (family Orchidaceae, official name “Shancigu” in the Chinese Pharmacopoeia 2025 Edition, verified by POWO) and the spike of *Prunella vulgaris* L. (family Lamiaceae, official name “Xiakucao” in the Chinese Pharmacopoeia 2025 Edition, verified by POWO), with species information shown in the verification Table 3.

Phlegm-heat binding is another key pattern in mammary hyperplasia, often seen in irritable patients with bitter taste and dry throat. This results from liver-fire condensing fluids into phlegm, forming nodules in the breast. The *Pseudobulbus Cremastrae seu Pleiones*-*Prunellae Spica* pair targets this precisely by resolving phlegm, dispersing nodules, and clearing liver-fire (Li et al., 2017; SptocagoCpmittod, 2022). When formulated with other botanical drugs, this combination exhibits therapeutic effects of resolving liver *qi* stagnation, regulating the *Chong* and *Ren* meridians, and dissolving phlegm accumulation in treating HMG caused by liver *qi* stagnation and *Chong*- *Ren* meridian dysfunction (Gong and Zhao, 2002). Modern pharmacological research indicates that *Pseudobulbus Cremastrae seu Pleiones* extract inhibits tumor cell proliferation and metastasis through multiple pathways (Ren and iuL, 2025). *Prunellae Spica* demonstrates multiple effects including endocrine regulation, antioxidant activity, and immune function enhancement (Li et al., 2019). The combined use of these two botanical drugs not only yields significant therapeutic effects for HMG—alleviating clinical symptoms such as breast distension, pain, and lumps—but also reduces the risk of malignant transformation by inhibiting tumor cell proliferation and metastasis. This holds crucial clinical value for improving patient quality of life and preventing disease progression.

Xie (Zhang et al., 2025) identified quercetin, β-sitosterol, stigmasterol, luteolin, and 2-methoxy-9,10-dihydro-4,5-dihydroxyphenanthrene-4,5-diol as the primary active metabolites in the combination of *Pseudobulbus Cremastrae seu Pleiones* and *Prunellae Spica* for treating HMG through network pharmacology analysis. This therapeutic mechanism may involve actions on key targets such as AKT1, TNF, IL-6, TP53, IL-1β, Prostaglandin-Endoperoxide Synthase-2 (PTGS2), Estrogen Receptor 1 (ESR1), thereby regulating lipid metabolism and signaling pathways such as atherosclerosis, AGE-RAGE, TNF, and IL-17 to achieve therapeutic effects on mammary hyperplasia. Additionally, the combination of “*Pseudobulbus Cremastrae seu Pleiones*-*Prunellae Spica*” may treat HMG by alleviating oxidative stress, regulating estrogen levels, inhibiting angiogenesis, and reducing inflammatory responses, while also preventing the progression of HMG to some extent. Molecular docking validation has confirmed the strong binding activity between the components and targets, providing a scientific theoretical basis for the clinical application of this herb pair and subsequent experimental research.

### 2.7 Herbal pair (*Sparganii Rhizoma*-*Curcumae Rhizoma*)

This botanical drug pair comprises the tuber of *Sparganium stoloniferum* (Graebn.) Buch-Ham. ex Juz. (family Typhaceae, official name “Sanleng” in the Chinese Pharmacopoeia 2025 Edition, verified by MPNS) and the rhizome of *Curcuma phaeocaulis* Val. (family Zingiberaceae, official name “Ezhu” in the Chinese Pharmacopoeia 2025 Edition, verified by POWO), with species information shown in the verification Table 3.

For severe, treatment-resistant cases with hard phlegm-heat nodules, mild therapies are inadequate. The *Sparganii Rhizoma*-*Curcumae Rhizoma* pair offers the potent, mass-dissipating action required for these challenging conditions (Tang et al., 2021). Modern pharmacological research indicates that the shared anti-cancer mechanisms of *Sparganii Rhizoma*-*Curcumae Rhizoma* include inducing tumor cell apoptosis, interfering with tumor cell cycle progression, inhibiting tumor angiogenesis, and regulating the tumor microenvironment (Kou et al., 2017). Phytochemical analysis reveals *Curcumae Rhizoma’s* active metabolites primarily comprise curcuminoids, volatile oils (containing curcumol), and polysaccharides. (Du et al., 2022). Curcuminoids serve as the key material basis for its blood-activating and stasis-resolving effects, while Curcuma volatile oil is the core metabolite responsible for its antitumor activity (Li et al., 2021), demonstrating potential applications in breast cancer prevention and treatment (Liu et al., 2022). Additionally, the total flavonoids in *Sparganii Rhizoma* have been confirmed to possess antitumor activity and anti-thrombotic effects, further expanding the clinical application scope of this medicinal botanical drug (Liu et al., 2021).

Zhang and colleagues (Zhang et al., 2023) compiled clinical protocols for treating HMG using the “*Sparganii Rhizoma*-*Curcumae Rhizoma*” combination. Based on literature analysis, the commonly used dosage for this combination in clinical treatment ranges from 6 to 20 g, typically administered in a 1:1 ratio. The development of HMG is often associated with *qi* stagnation, which subsequently leads to the formation of pathological products such as phlegm-dampness and blood stasis. As the condition progresses, symptoms of heat transformation may emerge. Therefore, treatment frequently employs botanical drugs that regulate *qi*, resolve phlegm, and clear heat, demonstrating therapeutic efficacy for symptoms like hardened masses, fever, and pain (Liu et al., 2007). In topical herbal formulations for HMG, some regimens incorporate borneol. Beyond its heat-clearing and pain-relieving properties, borneol opens the pores to penetrate directly through the skin to the affected area, enhancing drug absorption. This makes it a common metabolite in external treatments for HMG (Li et al., 2020). Within the strategy of modifying formulas based on individual symptoms, the “*Sparganii Rhizoma*-*Curcumae Rhizoma*” botanical drug pair is primarily applied for conditions characterized by persistent, hard breast masses that do not resolve, significant distending pain, severe blood stasis, robust constitution, and prolonged disease course (Cai, 2015; Guo, 2011; He and Wang, 2007; Liu, 2012; Jiang and Liu, 2007). The pharmacological distinction between *Sparganii Rhizoma* and *Curcumae Rhizoma* lies in their respective specialties: *Sparganii Rhizoma* excels at breaking blood stasis, while *Curcumae Rhizoma* excels at breaking *qi* stagnation. Their combined use enhances the efficacy of breaking blood stasis, promoting *qi* circulation to eliminate accumulation, and relieving pain. Therefore, for patients with more severe HMG (e.g., large, hard nodules with significant stasis), the “*Sparganii Rhizoma*-*Curcumae Rhizoma*” combination is a more suitable choice (Ye, 2003).

### 2.8 Herbal pair (*Fructus Ponciri trifoliatae*-*Gecko Chinensis*)

This herb pair is composed of the immature fruit of *Poncirus trifoliata* (L.) Raf. (family Rutaceae, official name “Gouju” in the Chinese Pharmacopoeia 2025 Edition, verified by POWO) and the whole body of *Gekko gecko* (Linnaeus) (family Gekkonidae, official name “Bihu” in the Chinese Pharmacopoeia 2025 Edition, verified by Reptile Database), with species information shown in the verification Table 3.

For mild cases with soft phlegm-qi nodules, gentle therapy is sufficient. The *Fructus Ponciri trifoliatae*-*Gecko Chinensis* pair provides targeted, moderate action to regulate qi and soften hardness, making it a specific choice for this condition. Unripe Citrus medica fruit, belonging to the Rutaceae family, possesses effects of soothing the liver and harmonizing the stomach, regulating *qi* to relieve pain, and eliminating food stagnation (Zhang et al., 2012). Its primary metabolites include flavonoids such as naringin and hesperidin, along with volatile oils, exhibiting pharmacological activities including antitumor and antioxidant properties (Li et al., 2016). *Gecko*, also known as Tianlong or Shougong (Tao, 2013; Liu et al., 2015), possesses properties to calm convulsions, subdue wind, soften hardened masses, disperse nodules, detoxify, and eliminate masses. Professor Jin *Qi*ngjiang specializes in using these two herbs to treat conditions characterized by accumulation, such as HMG, scrofula, goiter, and cancer (Wang, 2017). He posits that accumulation represents tangible disease, while mass formation represents intangible disease, each belonging to the categories of *qi* and blood respectively. Citrus aurantium excels at regulating and promoting *qi* flow, with its efficacy leaning toward *qi* movement, making it adept at treating accumulation syndromes. As a reptile, the *gecko* is inherently lively and, being a blood-and-flesh substance, belongs to the blood category, excelling at treating mass syndromes. The two metabolites complement each other, jointly acting on *qi* and blood to promote *qi* movement, resolve stagnation, soften hard masses, and eliminate masses. Their therapeutic effects are remarkable, and clinical application often yields excellent results.

Gu et al. (2021) employed network pharmacology methods to investigate metabolites in *Fructus Ponciri trifoliatae* such as bergapten, d-limonene, neohesperidin, and hesperidin, as well as amino acids in geckos. The study revealed that these metabolites interact with targets including ER, AR, CYP19A1, PTGS2, and CCND1 to jointly regulate the prolactin signaling pathway, vascular endothelial growth factor (VEGF) signaling pathway, and estrogen signaling pathway. It is thus inferred that the combination of “*Fructus Ponciri trifoliatae*-*Gecko Chinensis*” may directly or indirectly inhibit prolactin expression by affecting the expression of relevant genes in the PRL-PRLR downstream pathway, thereby suppressing mammary tissue hyperplasia. Additionally, the VEGF signaling pathway plays a crucial role in tumor angiogenesis, immune regulation, and direct effects on tumor cells. Inhibiting VEGF or other targets within this pathway can effectively curb tumor progression. At the molecular mechanism level: Bergapten from *Fructus Ponciri trifoliatae* depletes estrogen receptor (ER) through SMAD4-mediated ubiquitination, while d-limonene modulates cytochrome P450 to influence estrogen metabolism. Amino acids such as tyrosine and inosine in gecko contribute to regulating hormone receptor activity. These components collectively achieve a therapeutic effect characterized by “multi-component, multi-target, multi-pathway” interactions.

This study provides a scientific basis for the clinical application of the *Fructus Ponciri trifoliatae*-Gecko herb pair. However, limitations remain—such as the lack of incorporation of component content and pharmacokinetic parameters, as well as insufficient molecular-level characterization of gecko-derived polypeptides and polysaccharides. Subsequent research should integrate *in vitro* cellular experiments and animal models to validate the core mechanisms, thereby promoting standardized application in treating Hyperplasia of Mammary Glands (HMG).

## 3 Application of herbal pairs in prescriptions for treating HMG

This review systematically organizes the application of eight pairs of drugs for treating HMG in both classical formulas and clinical preparations, summarizing the crucial role of drug combinations in enhancing therapeutic efficacy, reducing toxicity, and achieving precision medication.

### 3.1 Utilization profiles and combination patterns of herbal pairs in classical and clinical formulations for HMG

**FIGURE 1:**
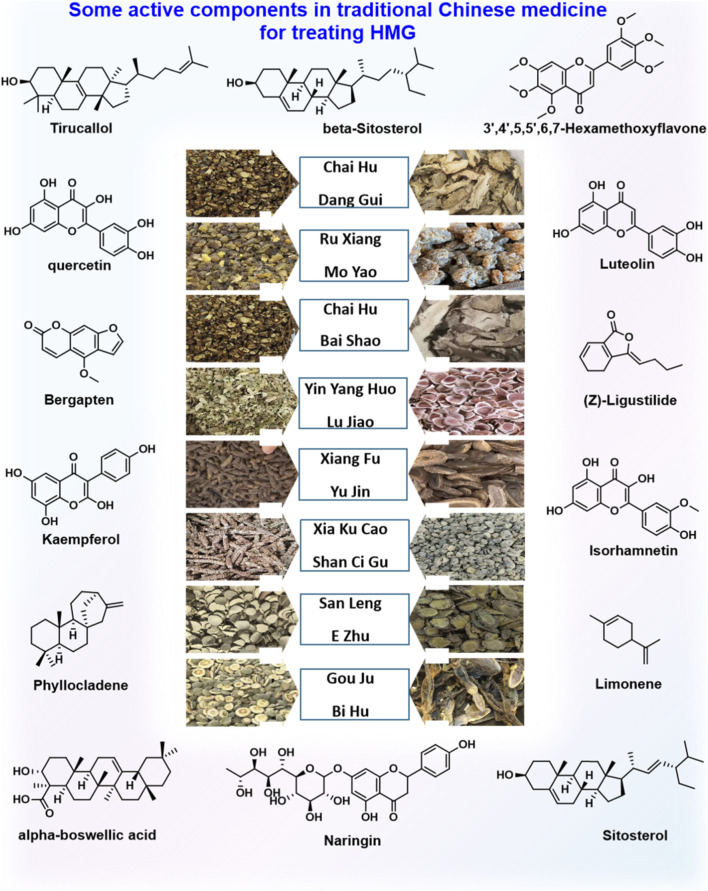
Some active metabolite in traditional Chinese medicine for treating HMG.

In traditional Chinese medicine formulas for treating HMG, common drug pairings include “*Angelicae Sinensis Radix* (Danggui) - *Bupleuri Radix* (Chaihu)”, “*Frankincense* (Ruxiang) - *Myrrh* (Moyao)”, and “*Curcumae Rhizoma* (E Zhu) - *Sparganii Rhizoma* (Sanling)”. Based on this analysis, the therapeutic approach for HMG centers on blood-activating and stasis-resolving herbs, supplemented by exterior-releasing agents, *qi*-regulating herbs, and heat-clearing herbs. This forms multiple combination patterns such as “blood-activating + exterior-releasing,” “blood-activating + *qi*-regulating,” and “blood-activating + heat-clearing.” (Figure 1). Research indicates that in the internal treatment of HMG, the primary therapeutic principles involve soothing the liver and resolving depression, as well as harmonizing the *Chong* and *Ren* meridians. Commonly used herbs include *Bupleuri Radix*, *Angelica*, *Cyperus*, and *Paeonia*, with *qi*-regulating herbs as the mainstay, supplemented by blood-activating and stasis-resolving agents, phlegm-resolving and cough-relieving agents, and tonifying herbs (Chen et al., 2021). In the external treatment of HMG, the main therapeutic approach is to promote blood circulation and remove blood stasis, with a relatively high proportion of blood-activating drugs used. At the same time, it is combined with drugs that relieve the exterior, clear heat, and regulate *qi* (Tan et al., 2020).

**FIGURE 2:**
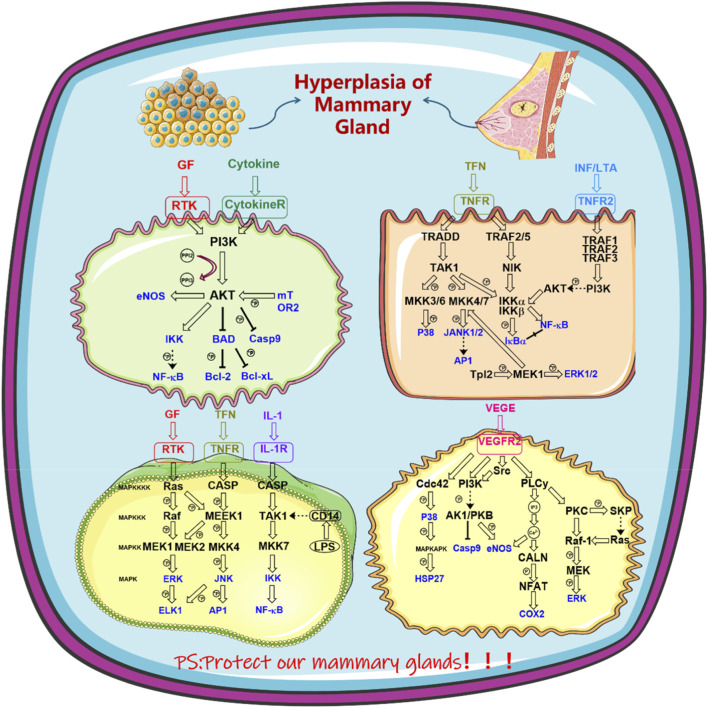
Herbal pairs therapy for HMG signaling pathway diagram.

The “*Angelicae Sinensis Radix* (Danggui) - *Bupleuri Radix* (Chaihu)” herb pair, as a metabolite of the renowned formula Xiaoyao Powder, has been shown through network pharmacology research to exert therapeutic effects on HMG by regulating IL-7 signaling pathways and endocrine resistance through modulating targets such as protein kinase and interleukin-6 (Yuan et al., 2022). The combination of “*Frankincense* (Ruxiang) - *Myrrh* (Moyao)” possesses blood-activating, stasis-resolving, and analgesic effects. Medical Synopsis Integrating Chinese and Western Medicine indicates that their pairing facilitates organ function and meridian circulation, not only unblocking meridian *qi* and blood but also regulating stagnation in organ *qi* and blood (Yang et al., 2024). The clinical combination of “*Curcumae Rhizoma* (E Zhu) - *Sparganii Rhizoma* (Sanling)” serves as a commonly used pair for promoting blood circulation and resolving stasis. Acorus excels at breaking blood stasis, while Curcuma focuses on promoting *qi* circulation. Research confirms that the active metabolites in this combination exert effects such as promoting blood circulation and unblocking meridians, inhibiting cell proliferation, and promoting apoptosis, elucidating the mechanism of their synergistic action (Xie, 2009) (Figure 2).

### 3.2 Core mechanisms of TCM herbal pairs in treating hyperplasia of mammary glands (HMG): Integrated interpretation with modern pharmacology

The scientific basis of Traditional Chinese Medicine (TCM) herbal pairs for treating HMG can be clearly elaborated through the deep integration of “regulation of core pathological pathways” and “core concepts of modern pharmacology.” On one hand, herbal pairs directly target the essence of HMG pathogenesis by synergistically intervening in four core pathological pathways: hormone regulation, inflammatory response, proliferation-apoptosis balance, and oxidative stress. On the other hand, these mechanisms are highly consistent with core modern pharmacological concepts such as receptor regulation, apoptosis modulation, and angiogenesis inhibition, forming an integrated therapeutic model characterized by “multi-component, multi-target, multi-pathway, and multi-pharmacological effects (Table 4).” Details are as follows:

**TABLE 4:** Molecular and cellular mechanisms of Chinese herbal pairs in treating hyperplasia of mammary gland (HMG).

Core functional pathways	Chinese herbal pairs	Key active metabolites	Target molecules/Signaling axes	Specific cellular and molecular effects
Hormone Regulation Pathway	*Radix Bupleuri-Angelicae Sinensis Radix*	Quercetin, Kaempferol, Z-ligustilide, Bornyl Acetate	Cytochrome P450 19A1, ERα	1. Downregulates ovarian E2 synthetase, significantly reduces serum E2 and PRL concentrations in HMG model rats (P < 0.05), and increases P4 levels; 2. Competitively binds to ERα in breast tissue, inhibits ERα-mediated CyclinD1 expression, and reduces hormone-dependent cell proliferation
	*Boswelliae Resina-Commiphorae Myrrhae Resina*	Boswellic Acid, β-Elemene, Acetyl-α-boswellic Acid, *Myrrh*ol	AR, ERβ, ERα	1. Regulates AR/ERβ balance to adjust the ratio of estrogen and androgen; 2. Downregulates ERα mRNA level in breast tissue (reduced by 40%–60% compared with the control group), blocks the transcriptional activation of proliferation genes by E2-ERα complex, and reduces abnormal differentiation of breast epithelial cells
	*Epimedii Folium-Cervi Cornu Pantotrichum*	Icariin, Deer *Antler* Extract, Deer *Antler* Polypeptide	PR, Pituitary PRL Secretion-Related Pathways	1. Icariin upregulates PR expression in breast tissue and enhances the inhibitory effect of P4 on proliferation; 2. Deer *Antler* extract inhibits pituitary PRL secretion, reduces serum PRL level, and rectifies the “estrogen excess and progesterone deficiency” imbalance
	*Cyperi Rhizoma-Curcumae Radix*	Isorhamnetin, Curcumin	ERα (ESR1), PR (PGR), FoxO3a	1. Inhibits ERα nuclear translocation and blocks hormone signal transduction; 2. Activates PR-mediated Bax expression, and simultaneously upregulates FoxO3a to promote Bax/FasL transcription, realizing bidirectional regulation of hormone signals
Inflammation Regulation Pathway	*Radix Bupleuri-Angelicae Sinensis Radix*	Quercetin, Kaempferol, Z-ligustilide, Bornyl Acetate	TNF Signaling Pathway, IL-7 Signaling Pathway, IKK/NF-κB p65, PTGS2	1. Downregulates the protein expression of TNF-α and IL-6 in breast tissue; 2. Inhibits IKK activity, prevents NF-κB p65 nuclear translocation, blocks PTGS2 transcription, reduces PGE2 synthesis, and alleviates breast distending pain
	*Boswelliae Resina-Commiphorae Myrrhae Resina*	Acetyl-α-boswellic Acid, *Myrrh*ol	NF-κB p65, TLR4/TRAF6, IL-1β/IL-17	1. Acetyl-α-boswellic acid directly binds to NF-κB p65 and inhibits its DNA-binding activity; 2. Downregulates TLR4/TRAF6 signaling, reduces the release of IL-1β and IL-17, decreases macrophage infiltration in breast tissue (by more than 30%), and alleviates fibrosis
	*Rhizoma Sparganii-Rhizoma Curcumae*	Curcumol, Total Flavonoids of Rhizoma Sparganii	MAPK (p38/JNK), NF-κB, IL-6/STAT3	1. Inhibits MAPK pathway phosphorylation and indirectly blocks NF-κB activation; 2. Reduces serum IL-6 level, inhibits IL-6/STAT3-mediated cell proliferation, and alleviates inflammation-related hyperplasia
	*Cremastrae Pseudobulbus Pleiones Pseudobulbus-Pruni Spinosae Spica*	β-Sitosterol, Luteolin, Ursolic Acid, Cremastrine Phenanthrene metabolites	AGE-RAGE Pathway, IL-17 Pathway, Th17 Cells	1. Reduces the stimulation of AGEs on breast tissue; 2. Inhibits IL-17 secretion by Th17 cells and blocks the inflammation-proliferation positive feedback loop; 3. Ursolic acid directly scavenges ROS and inhibits XO activity to reduce ROS production
Cell Proliferation/Apoptosis Pathway (PI3K/Akt Pathway Inhibition)	*Radix Bupleuri-Angelicae Sinensis Radix*	Volatile Oil (Z-ligustilide, Bornyl Acetate)	PI3K/Akt (p-Akt), PTEN, Bcl-2/Bcl-xl	1. Significantly reduces p-Akt expression in breast tissue (reduced by more than 50%, P < 0.05) and inhibits Bcl-2/Bcl-xl activation; 2. Upregulates PTEN expression, blocks proliferation signals, and reduces cell entry into S phase
	*Radix Bupleuri-Atractylodis Macrocephalae Rhizoma*	Quercetin, Baicalin	PI3K (PIK3CA), Akt, HRAS, PI3K/Akt/mTOR	1. Inhibits the binding of PI3K to cell membrane and reduces Akt phosphorylation; 2. Downregulates HRAS expression and inhibits abnormal proliferation of breast epithelial cells; 3. Induces autophagy through PI3K/Akt/mTOR pathway to clear damaged cells
	*Rhizoma Sparganii-Rhizoma Curcumae*	Curcuminoids, Total Flavonoids of Rhizoma Sparganii	PI3K (p110α), Akt/mTOR, Ribosomal Protein S6	1. Curcuminoids directly bind to PI3K p110α and inhibit its kinase activity; 2. Downregulates Akt/mTOR signaling, reduces ribosomal protein S6 phosphorylation, and induces cell arrest in G0/G1 phase
Cell Proliferation/Apoptosis Pathway (MAPK Pathway Regulation)	*Boswelliae Resina-Commiphorae Myrrhae Resina*	Boswellic Acid, β-Elemene	ErbB (EGFR), MAPK (p-ERK1/2/p-p38), CyclinD1/CDK4/CDK6	1. Downregulates EGFR expression, inhibits MAPK pathway phosphorylation, and induces G2/M phase arrest of breast epithelial cells; 2. Downregulates the mRNA levels of CyclinD1, CDK4 and CDK6 (reduced by 40%–60%), and prevents cells from entering S phase from G1 phase
	*Epimedii Folium-Cervi Cornu Pantotrichum*	Icariin, Deer *Antler* Extract	MAPK (JNK/ERK1/2), Bax/Bad, Bcl-2	1. Icariin activates JNK signaling and promotes Bax/Bad expression; 2. Deer *Antler* extract inhibits ERK1/2 phosphorylation and reduces Bcl-2 expression; 3. Synergistically increases the Bax/Bcl-2 ratio (from 0.3 to 0.8, P < 0.05) and induces abnormal cell apoptosis
Cell Proliferation/Apoptosis Pathway (Apoptosis-Related Protein Regulation)	*Cremastrae Pseudobulbus Pleiones Pseudobulbus-Pruni Spinosae Spica*	2-Methoxy-9,10-dihydro-4,5-dihydroxyphenanthrene-4,5-diol, Cremastrine Phenanthrene metabolites	Caspase-3/Caspase-9, Bcl-2, Mitochondrial Membrane Potential	1. Upregulates Caspase-3/Caspase-9 expression and activates the intrinsic apoptosis pathway; 2. Downregulates Bcl-2, increases the apoptosis index (AI) of breast tissue (from 5% to 15%, P < 0.05); 3. Stabilizes mitochondrial membrane and reduces cytochrome C release
	*Cyperi Rhizoma-Curcumae Radix*	Isorhamnetin, Curcumin	FoxO3a, Bax/FasL, Ki-67	1. Upregulates FoxO3a expression and promotes its nuclear translocation, transcriptionally activating Bax/FasL; 2. Inhibits the expression of proliferation protein Ki-67 (positive rate reduced by 30%) and induces breast epithelial cell apoptosis
Oxidative Stress Pathway	*Radix Bupleuri-Angelicae Sinensis Radix*	Quercetin, Kaempferol, Volatile Oil	SOD, GSH-Px, MDA	1. Significantly increases serum SOD and GSH-Px activities (increased by 40%–50% compared with the control group, P < 0.05); 2. Reduces the concentration of lipid peroxidation product MDA (reduced by more than 35%) and decreases oxidative damage to breast tissue
​	*Epimedii Folium-Cervi Cornu Pantotrichum*	Icariin, Deer *Antler* Polypeptide	Nrf2/ARE, HO-1/NQO1, Mitochondrial ROS	1. Icariin activates Nrf2/ARE signaling and upregulates HO-1 and NQO1 expression; 2. Deer *Antler* polypeptide inhibits mitochondrial ROS production, reduces the decrease of mitochondrial membrane potential, and decreases oxidative stress-induced DNA damage
	*Cremastrae Pseudobulbus Pleiones Pseudobulbus-Pruni Spinosae Spica*	Ursolic Acid, Cremastrine Phenanthrene metabolites	ROS, XO, Mitochondrial Membrane	1. Ursolic acid directly scavenges ROS and inhibits XO activity to reduce ROS production; 2. Cremastrine phenanthrene metabolites stabilize mitochondrial membrane, avoid excessive apoptosis induced by oxidative stress, and protect normal cells

#### 3.2.1 Receptor regulation: precise targeted intervention in hormone signaling

Consistent with modern receptor modulation theory, TCM herbal pairs regulate hormone balance via dual mechanisms. Key active metabolites (e.g., Z-ligustilide, quercetin in *Bupleuri Radix-Angelicae Sinensis Radix*) competitively bind to ERα/PR, blocking estrogen-mediated proliferation signals. Meanwhile, metabolites in *Cyperi Rhizoma-Curcumae Radix* inhibit CYP19A1 (aromatase), reducing peripheral estradiol (E2) synthesis. *Epimedii Folium-Cervi Cornu Pantotrichum* further modulates the E2/progesterone (P4) ratio, harmonizing the hypothalamic-pituitary-ovarian (HPO) axis.

#### 3.2.2 Apoptosis modulation: multi-link repair of proliferation-apoptosis balance

Herbal pairs correct dysregulated cell cycle and apoptosis through core signaling pathways. *Sparganii Rhizoma-Curcumae Rhizoma* inhibits PI3K/Akt/mTOR phosphorylation, inducing G0/G1 phase arrest. *Boswelliae Resina-Commiphorae Myrrhae Resina* downregulates CyclinD1/CDK4/CDK6, triggering G2/M phase block and promoting apoptosis via Bax/Bcl-2 ratio modulation. *Bupleuri Radix-Atractylodis Macrocephalae Rhizoma* activates PTEN, suppressing anti-apoptotic signals and clearing pathological cells.

#### 3.2.3 Inflammation regulation: systematic intervention in the NF-κB/cytokine network

Aligning with modern anti-inflammatory pharmacology, herbal pairs mitigate chronic inflammation. Acetyl-α-boswellic acid in Boswelliae Resina-Commiphorae *Myrrh*ae Resina directly binds NF-κB p65, inhibiting its nuclear translocation and reducing IL-6/TNF-α/COX-2 expression. *Pseudobulbus Cremastrae seu Pleiones*-*Prunellae Spica* downregulates TNF/IL-1β, alleviating inflammatory infiltration in hyperplastic tissues.

#### 3.2.4 Oxidative stress intervention: enhancement of antioxidant defense system

Herbal pairs combat oxidative damage by reinforcing antioxidant systems. Volatile oils and flavonoids in *Bupleuri Radix*-*Angelicae Sinensis Radix* upregulate SOD/GSH-Px activities, reducing malondialdehyde (MDA) levels. *Epimedii Folium*-Cervi Cornu Pantotrichum activates the Nrf2/ARE pathway, while *Cyperi Rhizoma*-*Curcumae Radix* inhibits NOX4, suppressing excessive ROS production.

#### 3.2.5 Angiogenesis inhibition: targeted improvement of the pathological microenvironment

Abnormal angiogenesis is suppressed via key pathway intervention. Curcumin and curcumol in *Sparganii Rhizoma*-*Curcumae Rhizoma* downregulate VEGFA, inhibiting endothelial cell proliferation. *Pseudobulbus Cremastrae seu Pleiones-Prunellae Spica* inhibits angiogenesis by reducing VEGF/bFGF levels, which may limit the nutrient supply to hyperplastic lesions and potentially lower the risk of malignant transformation—though further clinical evidence is needed to confirm the latter effect.

The mechanisms of TCM herbal pairs for treating HMG correspond precisely to core modern pharmacological concepts: receptor regulation corresponds to hormone signal modulation, apoptosis modulation corresponds to the repair of proliferation-apoptosis balance, inflammation regulation corresponds to the intervention in the NF-κB/cytokine network, oxidative stress intervention corresponds to the enhancement of antioxidant defense, and angiogenesis inhibition corresponds to the improvement of the pathological microenvironment. This integration not only reveals the combined regulatory of TCM herbal pairs as “multi-component, multi-target, and multi-pathway” but also clarifies their scientific connotation through modern pharmacological language. Unlike the limitations of single-target intervention in Western medicine, TCM herbal pairs can complement Western medicine treatments (e.g., alleviating inflammation and oxidative stress side effects of hormonal therapy), providing a solid theoretical basis for the integrated traditional Chinese and Western medicine treatment of HMG.

### 3.3 Discussion on clinical and preclinical study types of TCM herbal pairs for hyperplasia of mammary glands (HMG)

The therapeutic effects and mechanisms of TCM herbal pairs for HMG have been verified by multiple study types, including preclinical studies, observational clinical studies, and randomized controlled trials (RCTs). Each type has unique advantages in evidence generation, as well as inherent limitations. A systematic review of their characteristics is crucial for evaluating the credibility of research findings and guiding future research directions.

#### 3.3.1 Preclinical studies

Preclinical studies, centered on *in vitro* cell experiments, *in vivo* animal models, and network pharmacology analyses, lay the foundation for clarifying molecular mechanisms and guiding clinical applications.

Advantages: They allow precise control of experimental conditions to explore mechanisms at the cellular and molecular levels. For example, network pharmacology has identified the core metabolites (quercetin, kaempferol) and key targets (AKT1, IL-6, etc.) of the *Bupleuri Radix*-*Angelicae Sinensis Radix* pair; *in vitro* experiments have confirmed that the Frankincense-*Myrrh* pair exerts a dose-dependent inhibitory effect on the proliferation of estradiol-induced mammary cells; and animal experiments have verified that *Bupleuri Radix*-*Angelicae Sinensis Radix* can regulate serum hormone levels and enhance antioxidant activity in model rats.

Limitations: Animal models (e.g., estradiol-induced rats) cannot fully simulate the complex pathological environment of human HMG (such as emotional stress and cyclic hormone fluctuations); network pharmacology predictions rely on the completeness of databases, and some targets require experimental validation; there are differences in biological characteristics between *in vitro* cell lines and primary mammary epithelial cells.

#### 3.3.2 Observational clinical studies

Observational clinical studies, including retrospective analyses of clinical prescriptions, case series, and real-world studies, focus on the practical clinical application of herbal pairs and reflect their effectiveness in a broad population.

Advantages: They align with real-world clinical scenarios. For instance, an analysis of HMG-related patents and prescriptions revealed that *Bupleuri Radix*-*Angelicae Sinensis Radix* and Frankincense-*Myrrh* are the most commonly used herbal pairs—the former is often modified based on Xiaoyao Powder to adapt to different TCM syndromes, while the latter has the highest frequency of application in external treatments.

Limitations: Confounding factors (e.g., concurrent use of Western drugs, differences in lifestyle) exist, making it difficult to isolate the efficacy of the herbal pair itself; the lack of randomization and control groups results in lower evidence quality compared to RCTs.

#### 3.3.3 Randomized controlled trials (RCTs)

Due to their rigorous design (randomization, blinding, and control groups), RCTs are regarded as the gold standard for evaluating therapeutic efficacy and provide high-level evidence for the clinical application of herbal pairs.

Advantages: They have a low risk of bias and reliable efficacy verification. For example, modified Xiaoyao Powder containing *Bupleuri Radix*-*Angelicae Sinensis Radix* can significantly reduce serum estradiol levels and improve breast pain in young female HMG patients; Xihuang Pills containing Frankincense-*Myrrh* have a total effective rate of over 85% in treating HMG, which is superior to tamoxifen.

Limitations: Most existing studies have small sample sizes (mostly <100 cases), short follow-up periods (mostly <1 year), and inconsistent protocols. Due to differences in TCM syndrome differentiation, the dosages and modifications of herbal pairs vary, making it difficult to standardize RCT protocols.

Table 5 summarizes core methodological and outcome details for pharmacological studies (encompassing both *in vitro* cell experiments and *in vivo* animal models), such as extract types, minimum effective concentrations/doses, and control settings. Table 6 focuses on clinical studies, detailing intervention protocols, subject characteristics, follow-up durations, and efficacy outcomes to clarify the real-world application and evidence quality of herbal pairs.

**TABLE 5:** Summary of key information for pharmacological studies (in vitro/in vivo).

Herbal pair	Study type	Extract type	Model	Dose/Concentration range	Minimum effective concentration (MEC)/Dose (MED)	Control setting	Duration	Ref.
*Bupleuri Radix-Angelicae Sinensis Radix*	*In vivo* (animal)	Volatile oil + ethanol extract	Estradiol-induced HMG rat model (Sprague-Dawley rats)	Volatile oil: 100–200 mg/kg (gavage)	100 mg/kg (significantly downregulates serum E2)	Positive: Tamoxifen (1 mg/kg, gavage); Negative: Normal saline	4 weeks	Ding et al. (2025), Kim and Ko (2021)
*Frankincense-Myrrh*	*In vitro* (cell)	Ethanol extract	Estradiol-induced human mammary epithelial cell model (MCF-10A)	50–200 μg/mL (culture medium)	50 μg/mL (proliferation inhibition rate >30%)	Positive: Tamoxifen (10 μM); Negative: DMSO	48 h	Deng et al. (2023)
*Frankincense-Myrrh*	*In vivo* (animal)	Aqueous extract	Estradiol + progesterone-induced HMG rat model	200–400 mg/kg (gavage)	200 mg/kg (downregulates CyclinD1 mRNA by 40%)	Positive: Tamoxifen (1 mg/kg); Negative: Normal saline	3 weeks	Gong et al. (2024)
*Bupleuri Radix-Atractylodis Macrocephalae Rhizoma*	*In vivo* (animal)	Aqueous extract (drug-containing serum of Xiaoyao Powder)	Normal mouse + HMG mouse model	Drug-containing serum: 10%–20% (*in vitro*, medium); Gavage: 15 g/kg (mouse)	10% drug-containing serum (inhibits PI3K/Akt phosphorylation)	Negative: Blank serum/normal saline	*In vitro*: 24 h; *In vivo*: 2 weeks	Zhang and Lu (2013), Wu et al. (2019a)
*Epimedii Folium-Antler*	*In vivo* (animal)	Icariin + *Antler* polypeptide	Ovariectomized HMG rat model	Icariin: 20–50 mg/kg; *Antler* polypeptide: 10–30 mg/kg (both gavage)	Icariin 20 mg/kg (upregulates PR expression)	Positive: Estradiol (0.1 mg/kg); Negative: Normal saline	5 weeks	Xu and Wang (2006), Wu et al. (2013)
*Sparganii Rhizoma-Curcumae Rhizoma*	*In vitro* (cell)	Curcumol + total flavonoids of Sparganii	Primary cultured human mammary hyperplastic cell model	Curcumol: 10–50 μM; Total flavonoids: 50–150 μg/mL	Curcumol 10 μM (induces G0/G1 arrest)	Positive: Cisplatin (5 μM); Negative: DMSO	72 h	Kou et al. (2017), Liu et al. (2022)
*Pseudobulbus Cremastrae seu Pleiones-Prunellae Spica*	*In vitro* (cell)	Phenanthrenes (from *Pseudobulbus Cremastrae seu Pleiones*) + prunellin (from *Prunellae Spica*)	MCF-7 cell model (mimicking HMG proliferation)	Phenanthrenes: 5–20 μM; Prunellin: 25–100 μg/mL	Phenanthrenes 5 μM (inhibits IL-6 expression)	Negative: DMSO	36 h	Zhang et al. (2025)

**TABLE 6:** Summary of key information for clinical studies.

Herbal pair	Study type	Subjects	Intervention	Control setting	Duration	Efficacy indicators	Total effective rate/Outcome	Ref.
*Frankincense-Myrrh (external use)*	Retrospective Case Series	HMG patients (n = 45, aged 25–45 years)	*Frankincense*-*Myrrh ointment* (1:1 ratio, 20 g/tube), external application on mammary lumps, twice daily	Self-controlled (pre-post)	8 weeks	Pain score (VAS), lump diameter	82.2% (pain relief)	Wang (2015)
*Xihuang Pills (containing Frankincense-Myrrh)*	Randomized Controlled Trial (RCT)	HMG patients (n = 80, aged 22–48 years)	Xihuang Pills (0.2 g/pill), oral administration, 3 pills twice daily	Positive: Tamoxifen (10 mg/day)	12 weeks	Lump shrinkage rate, serum E2/PRL levels	85% vs. 65% (tamoxifen)	Tang et al. (2024), Ge et al. (2022)
*Bupleuri Radix-Angelicae Sinensis Radix (modified Xiaoyao Powder)*	Retrospective Case Series	Young female HMG patients (n = 60, <35 years)	Modified Xiaoyao Powder (*Bupleuri Radix* 6 g + *Angelicae Sinensis Radix* 10 g + other herbs), decoction, oral administration once daily	Self-controlled (pre-post)	6 weeks	Serum E2 level, frequency of breast distension/pain	78.3% (symptom improvement)	Zhao (2021)
*Sparganii Rhizoma-Curcumae Rhizoma (oral use)*	Clinical Observation	HMG patients with severe blood stasis (n = 38)	*Sparganii Rhizoma* 6–10 g + *Curcumae Rhizoma* 6–10 g (1:1 ratio), decoction, oral administration once daily	Positive: Rupixiao Tablets (5 tablets/time, 3 times daily)	10 weeks	Lump hardness score, blood stasis syndrome score	76.3% vs. 60.5% (Rupixiao Tablets)	Zhang et al. (2023) Jiang and Liu (2007)
*Cyperi Rhizoma-Curcumae Radix (Xiangfu Yujin Decoction)*	Case Series	HMG patients with liver qi stagnation (n = 52)	*Cyperi Rhizoma* 12 g + *Curcumae Radix* 10 g, decoction, oral administration once daily	Self-controlled (pre-post)	4 weeks	Breast distension relief time, serum EGFR level	75% (distension relief)	Li et al. (2022)

### 3.4 Comparative discussion on herbal pairs and existing therapies

The therapeutic paradigm for HMG in modern medicine is defined by its strategy of “single-target precision intervention,” concentrating on discrete mechanisms like hormonal pathways or proliferative signals. A key limitation of this model is its constrained scope, leading to incomplete management of the disease pathology. Chinese herbal pairs, operating through a “multi-component, multi-target, multi-pathway” framework, present a contrasting strategy. They simultaneously tackle broader pathological metabolites (e.g., inflammation, oxidative stress) and foundational TCM dysfunctions, thereby promoting global functional recovery. This fundamental distinction establishes a powerful therapeutic complementarity between the two paradigms (Table 7).

**TABLE 7:** Comparison of mechanisms and complementary relationships between Chinese herbal pairs and western medicine therapies for hyperplasia of mammary gland (HMG).

Comparison dimensions	Mechanistic characteristics of western medicine therapies for HMG	Mechanistic characteristics of Chinese herbal pairs	Complementary relationships
Core Intervention Model	Precise single-target intervention, focusing on local pathological processes (e.g., hormonal signaling or cell proliferation) with insufficient holistic regulation.	Multi-component, multi-target, and multi-pathway synergistic regulation, integrating local pathological improvement and holistic functional modulation based on TCM pathogenesis.	Synergy of “precise blocking + systematic regulation,” addressing the limitations of the slow onset of Chinese herbal pairs and the insufficient holistic intervention of Western medicine.
Hormonal Regulation Mechanism	Single-target blocking or modulation: e.g., Tamoxifen competitively binds to estrogen receptors (ER) on mammary gland cells to block estrogenic effects; bromocriptine inhibits prolactin secretion. However, it fails to intervene in inflammation or oxidative damage associated with hormonal imbalance.	Bidirectional synergistic regulation: e.g., *Chaihu-Dangui* (*Bupleurum chinense* DC.-*Angelica sinensis* (Oliv.) Diels) downregulates serum estradiol (E2) and prolactin (PRL) levels while upregulating progesterone (P4); *Yinyanghuo-Lurong* (*Epimedium brevicornu* Maxim.-Cervus elaphus L. velvet) modulates the hormonal axis and inhibits mammary fibrosis simultaneously.	Western medicine rapidly blocks core hormonal signals, while Chinese herbal pairs correct hormonal imbalance and promote tissue repair, mitigating the side effect of endocrine disorders associated with Western medicine.
Cell Proliferation Regulation	Only inhibits a single proliferation signal (e.g., prolactin-mediated proliferation) and cannot induce cell cycle arrest; surgical resection directly removes lesions but fails to eliminate the underlying causes (e.g., hormonal imbalance or signaling pathway dysregulation).	Multi-link regulation: e.g., *Ruxiang-Moyao* (Boswellia carteri Birdw.-*Commiphora Myrrha* (Nees) Engl.) induces G2/M phase cell cycle arrest; *Chaihu-Baizhu* (*Bupleurum chinense* DC.-*Atractylodes macrocephala* Koidz.) inhibits abnormal proliferation and promotes apoptosis.	Western medicine enables rapid lesion control, while Chinese herbal pairs reverse abnormal proliferation by regulating the cell cycle and apoptosis, thereby reducing the recurrence rate.
Inflammation and Oxidative Stress Intervention	Lacks targeted interventions, failing to ameliorate inflammation and oxidative damage associated with HMG.	Multi-target intervention: e.g., *Ruxiang-Moyao* modulates the levels of tumor necrosis factor (TNF) and interleukin-6 (IL-6) to inhibit inflammation; *Chaihu-Dangui* enhances the activities of superoxide dismutase (SOD) and glutathione peroxidase (GSH-Px), while reducing malondialdehyde (MDA) levels to alleviate oxidative damage.	Chinese herbal pairs fill this gap by targeting inflammatory and oxidative stress pathways, alleviating tissue damage at the pathological source which is not addressed by Western medicine.
Signaling Pathway Regulation	Single-point regulation, failing to achieve comprehensive regulation of core signaling pathways involved in HMG (e.g., PI3K/Akt and mTOR pathways).	Multi-pathway synergistic regulation: e.g., *Ruxiang-Moyao* modulates 6 pathways including steroid hormone synthesis and mTOR, downregulating CyclinD1 expression; *Chaihu-Dangui* reduces PI3K/Akt phosphorylation and regulates apoptotic protein expression.	Chinese herbal pairs complement the limited pathway regulation of Western medicine, achieving multi-level regulation at the pathway, molecular, and cellular levels.
Holistic Functional Modulation	Focuses solely on local pathological changes without improving the imbalance of qi, blood, and zang-fu organs in the body (core concepts in TCM).	TCM pathogenesis-targeted modulation: e.g., *Xiangfu-Yujin* (*Cyperus rotundus L.*-Curcuma aromatica Salisb.) soothes the liver and promotes blood circulation, relieving breast distension and correcting hormonal imbalance; *Zhebeimu-Xiakucao* (Fritillaria thunbergii Miq.-*Prunella vulgaris* L.) resolves phlegm and dissipates nodules, inhibiting inflammation and angiogenesis.	Western medicine addresses the symptoms while Chinese herbal pairs target the root causes by improving holistic function, realizing the integration of symptomatic treatment and etiological treatment.
Target Coverage Range	Narrow coverage, only involving a few targets such as estrogen receptor (ER) and progesterone receptor (PR).	Broad coverage, with a single herbal pair regulating dozens to hundreds of potential targets; e.g., *Ruxiang-Moyao* regulates 271 potential targets related to HMG.	Combination of “core target blocking” by Western medicine and “comprehensive pathological link coverage” by Chinese herbal pairs, forming a full-spectrum regulatory network.
Focus of Pathological Intervention	Acute symptom management: Drugs (e.g., analgesics) rapidly relieve pain, and surgery removes lesions, but neither reverses the pathological progression.	Pathological reversal: Achieves multi-dimensional reversal of pathological processes through “hormonal balance regulation, inflammation control, signaling pathway modulation, and TCM pathogenesis-targeted intervention.”	Western medicine provides rapid acute symptom control, while Chinese herbal pairs achieve in-depth pathological reversal and reduce recurrence after treatment cessation, achieving the synergistic effect of “acute symptom control + long-term stability.”

Notably, while the aforementioned herb pairs have demonstrated distinctive anti-hyperplasia mechanisms compared with conventional therapies, the reliability of their mechanistic conclusions varies significantly depending on the depth of experimental validation. Specifically, some studies have formed a complete evidence chain from metabolite identification to *in vivo*/*in vitro* functional verification, while others remain at the stage of network pharmacology prediction without experimental confirmation. To systematically clarify the credibility hierarchy of existing research results and provide clear references for subsequent studies, the following table classifies 8 classic herb pairs based on the level of experimental validation, and details their core evidence and potential limitations (Table 8).

**TABLE 8:** Grading table of relevance of effects of traditional Chinese medicine herb pairs in treating hyperplasia of mammary gland (HMG).

Herb pair	Relevance grade	Core validation basis	Limitations
*Bupleuri Radix-Angelicae Sinensis Radix* (including essential oil BAO)	Highly Relevant	1. 18 active metabolites identified by Gas Chromatography-Mass Spectrometry (GC-MS), with Z-ligustilide (39.24%) and decyl acetate (25.37%) as core metabolites; 2. *In vivo* (rat model): Significantly regulated serum estradiol, prolactin and progesterone levels, inhibited PI3K/Akt pathway (P < 0.05) with dose-dependent effects; 3. Molecular docking: Core metabolites bound to AKT1 and TNF with binding energy ≤−6.0 kcal/mol; 4. Supported by partial *in vitro* cell experiment data.	1. Anti-hyperplasia effect of non-volatile monomer metabolites (e.g., saikosaponin C) not verified; 2. Insufficient pharmacokinetic data in clinical samples.
*Frankincense-Myrrh*	Highly Relevant	1. 51 active metabolites identified, including boswellic acid and guggulsterone; 2. *In vitro* (HBL-100 cells): 20% drug-containing serum inhibited proliferation (inhibition rate higher than tamoxifen, P < 0.01), arrested cells at G_2_/M phase, and downregulated CyclinD1/CDK4/6; 3. *In vivo* (rat model): Reduced breast diameter and nipple height, downregulated ERα and CyclinD1 expression in mammary tissue (P < 0.01); 4. Molecular docking: Quercetin bound to AKT1 and TP53 with binding energy ≤−7.0 kcal/mol.	1. Individual mechanism of monomer metabolites (e.g., mansumbinoic acid) not clarified; 2. *In vitro* experiments did not simulate the complete *in vivo* metabolic process.
*Pseudobulbus Cremastrae seu Pleiones-Prunellae*	Moderately Relevant	1. 13 active metabolites identified, including quercetin and β-sitosterol; 2. Molecular docking: Core metabolites bound to AKT1, TNF and TP53 with binding energy ≤−7.5 kcal/mol; 3. Pathway enrichment results consistent with the oxidative stress and inflammation mechanisms of mammary gland hyperplasia.	1. No *in vivo*/*in vitro* functional experiments to verify the anti-hyperplasia effect; 2. Actual content of core metabolites in the herb pair extract not detected; 3. Direct regulatory relationship of metabolite- target - pathway not confirmed.
*Bupleuri Radix-Atractylodis Macrocephalae Rhizoma*	Moderately Relevant	1. 17 active metabolites identified, including baicalin and quercetin; 2. Molecular docking: Core metabolites had good binding affinity with AKT1 and PRKCA (docking score ≥4.25); 3. Indirect evidence: Isorhamnetin and quercetin were confirmed to inhibit the proliferation of breast-related cells in other studies.	1. No direct animal/cell experiments to validate this herb pair; 2. Regulatory effect of core targets (e.g., PIK3CA) not verified in the mammary gland hyperplasia model; 3. Bioavailability of metabolites in *Atractylodis Macrocephalae Rhizoma* not clarified.
*Cyperi Rhizoma-Curcumae Radix*	Moderately Relevant	1. 33 active metabolites identified, including isorhamnetin and stigmasterol; 2. Protein-Protein Interaction (PPI) network identified core targets such as ESR1 and EGFR; Kyoto Encyclopedia of Genes and Genomes (KEGG) enrichment showed the PI3K/AKT pathway (consistent with the hormone imbalance mechanism); 3. Cross-evidence: Core metabolite effects were validated in other herb pairs.	1. No molecular docking or functional experiments to verify the overall anti-hyperplasia effect of the herb pair; 2. Actual existence and content of metabolites in the extract not identified by High-Performance Liquid Chromatography (HPLC)/GC-MS; 3. *In vivo* absorption and metabolism of metabolites not clarified.
*Epimedii Folium-Cervi Cornu*	Moderately Relevant	1. 34 active metabolites identified, including quercetin and luteolin; 2. Molecular docking: Core metabolites bound to TP53 and AKT1 with binding energy ≤−5.0 kJ/mol; 3. Indirect evidence: Icariin regulated the estrogen-progesterone ratio; Cervi Cornu extract reduced serum estradiol levels in other studies.	1. Pathological improvement effect on mammary gland hyperplasia alone not verified (experiments focused on “hyperplasia + anxiety”); 2. Regulatory effect of core pathways (e.g., FoxO, HIF-1) not confirmed in mammary tissue; 3. Synergistic mechanism of herb pair compatibility not clarified.
*Ponciri Trifoliatae Fructus-Gekko*	Lowly Relevant	1. 29 metabolites predicted by network pharmacology (18 from Ponciri Trifoliatae Fructus, 24 small-molecule amino acids/nucleosides from Gekko); 2. Target mapping obtained potential targets such as ER, AR and CYP19A1; enriched prolactin, estrogen and VEGF signaling pathways.	1. No molecular docking or *in vivo*/*in vitro* functional validation; 2. Actual existence and content of predicted metabolites in the extract not experimentally identified; 3. Gekko components only studied as “small-molecule amino acids” with unclear biological activity; 4. Component-target-pathway association only computer-simulated.

### 3.5 Discussion on research limitations and future directions

#### 3.5.1 Research limitations

Although current studies on Traditional Chinese Medicine (TCM) herbal pairs for treating Hyperplasia of Mammary Glands (HMG) have initially revealed their multi-target and multi-pathway mechanisms, there remain four critical shortcomings that restrict their clinical translation and in-depth mechanistic exploration:

Lack of quantitative verification of complementary regulatory effects: Most studies only qualitatively describe the “complementary regulatory effects” of herbal pairs (e.g., the *Bupleuri Radix*-*Angelicae Sinensis Radix* pair “soothes the liver without consuming liver blood and nourishes blood without stagnating liver qi”). However, they fail to quantify complementary regulatory effects using pharmacological indicators such as the Combination Index (CI) or isobologram analysis. For instance, the synergistic ratio between quercetin (from *Bupleuri Radix*) and Z-ligustilide (from *Angelicae Sinensis Radix*) has not been clarified, nor has the difference in proliferation inhibition rates between “herbal pairs vs. single herbs” been compared, making it impossible to define the specific contribution of complementary regulatory effects.

Insufficient quality of clinical evidence: Clinical studies are predominantly small-sample (≤100 cases) retrospective case series (e.g., a study on external application of the Frankincense-*Myrrh* pair with n = 45, and a study on modified Xiaoyao Powder containing *Bupleuri Radix*-*Angelicae Sinensis Radix* with n = 60). Only one study was designed as a Randomized Controlled Trial (RCT), and its sample size was merely 80 cases. Additionally, the follow-up duration is generally <6 months, lacking data on recurrence rates over 1 year or longer, as well as safety monitoring of liver/kidney function and hormone axes during long-term medication (e.g., >6 months). The evidence level is insufficient to support recommendations in clinical guidelines.

Gaps in bioavailability and tissue distribution data: Active metabolites predicted by network pharmacology (e.g., bergapten and d-limonene from the *Fructus Ponciri trifoliatae*-Gecko pair, and β-sitosterol from the *Pseudobulbus Cremastrae seu Pleiones*-*Prunellae Spica* pair) have only been validated for target-binding affinity via molecular docking. Their concentration-time profiles in human mammary tissue and plasma have not been detected using techniques such as UPLC-Q-orbitrap-MS. This failure to confirm the critical link of “metabolites reaching the target site and exerting pharmacodynamic effects” results in a broken “component-target-efficacy” association.

Limited relevance of disease models to clinical conditions: Experimental studies mostly adopt a “short-term estradiol-induced” rat model of HMG (modeling cycle: 2–4 weeks), which only simulates the single pathogenesis of “excessive estrogen” and does not incorporate key factors of human HMG, such as chronic cyclic characteristics (e.g., alternating fluctuations of estrogen and progesterone during the menstrual cycle) and emotional stress (a core TCM pathogenesis of “liver qi stagnation”). This leads to a significant discrepancy between the model and the clinical pathological environment, reducing the translational value of mechanistic research.

#### 3.5.2 Future directions

To address the above limitations, future research should focus on five key breakthrough areas to advance the study of TCM herbal pairs for HMG toward precision and clinical applicability:

Conduct high-quality clinical studies: Prioritize the design of multi-center, double-blind, randomized controlled trials (RCTs) with a sample size >200 cases. Tamoxifen should be used as a positive control, and a placebo group should be included. The follow-up duration should be extended to 1–3 years to evaluate not only the short-term efficacy indicators (e.g., lump shrinkage rate, pain relief rate) of herbal pairs (e.g., *Sparganii Rhizoma*-*Curcumae Rhizoma*, Frankincense-*Myrrh*) but also long-term indicators such as recurrence rates and Ki-67 indices in mammary tissue. Meanwhile, safety indicators (e.g., liver/kidney function, endometrial thickness) should be monitored to improve the evidence level.

Quantify the synergistic mechanisms of herbal pairs: Based on *in vitro* cell models (e.g., MCF-10A mammary epithelial cells, primary human mammary hyperplastic cells), quantify the complementary regulatory effects of core metabolites using Combination Index (CI) assays. For example, verify the synergistic inhibition ratio of boswellic acid and β-elemene (from the Frankincense-*Myrrh* pair) and isorhamnetin and curcumin (from the *Cyperi Rhizoma*-*Curcumae Radix* pair). Combine “herbal pair decomposition experiments” (comparing herbal pairs, single herbs, and metabolite combinations) to clarify the functional weight of each metabolite in regulating pathways such as PI3K/Akt and ERα.

Supplement pharmacokinetic studies: Use UPLC-Q-orbitrap-MS to detect the concentrations of active metabolites in the plasma and mammary tissue of healthy volunteers or HMG patients after administration of herbal pairs. Analyze pharmacokinetic parameters such as absorption half-life (t_1_/_2_) and peak concentration (Cmax). For instance, clarify the targeted distribution efficiency of Z-ligustilide (from the *Bupleuri Radix*-*Angelicae Sinensis Radix* pair) in mammary tissue to optimize clinical dosage (e.g., 1 vs. 2 doses per day) and dosage forms (e.g., volatile oil nanoemulsions).

Optimize the construction of disease models: Develop HMG animal models that better mimic clinical conditions, such as a “chronic estrogen-progesterone alternating induction + restraint stress” rat model, which simulates both hormonal fluctuations during the human menstrual cycle and the TCM pathogenesis of “liver qi stagnation.” Alternatively, use transgenic mouse models (e.g., ERα-overexpressing mice) to more accurately replicate the molecular pathological characteristics of HMG and enhance the clinical relevance of mechanistic research.

Explore integrated TCM-Western medicine regimens: Conduct studies on the combined use of TCM herbal pairs and Western medicines. For example, evaluate the efficacy of the *Bupleuri Radix*-*Angelicae Sinensis Radix* pair combined with low-dose tamoxifen, verifying whether the herbal pair can reduce the risk of tamoxifen-induced endometrial hyperplasia by regulating estrogen metabolism (e.g., inhibiting CYP19A1) while enhancing lump shrinkage rates through synergistic inhibition of the PI3K/Akt pathway. This will form an integrated treatment regimen characterized by “rapid symptom control + long-term stability.”

### 3.6 Core contributions to the field

#### 3.6.1 Establish a “Pathogenesis-Syndrome-Herb Pair-Mechanism” precision matching system, addressing the clinical dilemma of syndrome-based medication

Hyperplasia Mammary Gland (HMG) presents diverse Traditional Chinese Medicine (TCM) syndromes (e.g., liver qi stagnation, blood stasis, phlegm coagulation, kidney deficiency), and existing studies predominantly report individual herb pairs in isolation, lacking systematic integration aligned with pathogenesis progression. For the first time, this review constructs a hierarchical framework sorted by disease course evolution: “early-stage qi stagnation (Cyperi Rhizoma-Curcumae Radix) → mid-stage phlegm-blood stasis (Boswelliae Resina-Commiphorae Myrrhae Resina, Cremastrae Pseudobulbus Pleiones Bulbus-Pruneilae Spica) → late-stage kidney deficiency/severe blood stasis (Epimedii Folium-Cervi Cornu, Sparganii Rhizoma-Curcumae Rhizoma)”. It further clarifies the precise correspondence between specific syndromes (e.g., liver stagnation with blood deficiency, liver stagnation with spleen deficiency, phlegm-heat stagnation) and herb pairs, resolving the long-standing lack of systematic theoretical support for “syndrome-guided herb pair selection” in clinical practice. This system provides a directly actionable roadmap for personalized MGH treatment.

#### 3.6.2 Bridge TCM compatibility theory with modern pharmacology, deciphering the holistic synergy of herb pairs

Current research often dissociates TCM compatibility principles (e.g., “mutual reinforcement” “mutual assistance”) from modern molecular mechanisms, reducing herb pairs to a list of isolated metabolites. For each herb pair, this review first elaborates its TCM compatibility logic (e.g., *Bupleuri Radix*-Angelicae Sinensis Radix “soothes the liver without consuming liver blood, nourishes blood without stagnating liver qi”), then systematically deciphers the synergistic material basis (e.g., saikosaponins from *Bupleuri Radix* and Z-ligustilide from Angelicae Sinensis Radix) and integrated molecular mechanisms (e.g., concurrent regulation of PI3K/Akt and estrogen signaling pathways). This is the first work to establish a direct link between TCM compatibility theory and multi-component synergism, providing a concrete scientific explanation for TCM’s “holistic view” and advancing herb pair research from “metabolites enumeration” to “synergistic logic dissection.”

#### 3.6.3 Construct a four-tier evidence chain to enhance translational credibility

Most existing reviews rely solely on network pharmacology predictions or single-center clinical observations, resulting in fragmented evidence. This review integrates a rigorous four-tier evidence chain for each herb pair: “network pharmacology target prediction → *in vitro* cell experiments (proliferation/apoptosis validation) → *in vivo* animal models (pathological/hormonal regulation) → clinical efficacy verification”. It emphasizes critical validation steps such as “drug-containing serum assays” and “compatibility decomposition experiments”, quantifies the contribution of core metabolites (e.g., boswellic acid content ≥5%), and confirms complementary regulatory effects through combination index analysis. This comprehensive evidence integration significantly enhances the reliability of mechanistic conclusions and lays a solid foundation for subsequent formulation development, dosage optimization, and clinical translation.

## 4 Conclusion

Hyperplasia of Mammary Glands (HMG), a prevalent gynecological disorder with potential malignant transformation risk, poses a significant threat to women’s health globally (Li et al., 2017; Yang et al., 2025). This review systematically synthesized the therapeutic mechanisms and clinical applications of eight classic Traditional Chinese Medicine (TCM) herbal pairs in treating HMG, integrating evidence from network pharmacology, molecular biology, preclinical experiments, and clinical studies. The findings collectively validate the scientific rationale of TCM herbal pair compatibility and highlight their unique advantages in addressing HMG’s complex pathogenesis.

First, the eight herbal pairs—including *Bupleuri Radix-Angelicae Sinensis Radix*, *Frankincense-Myrrh*, and *Sparganii Rhizoma-Curcumae Rhizoma*—exert combined therapeutic effects primarily through four core pathological pathways, which align closely with both TCM pathogenesis and modern medical insights. Specifically, they regulate estrogen/progesterone signaling to correct hormonal imbalance (e.g., *Bupleuri Radix-Angelicae Sinensis Radix* competitively binds estrogen receptor α (ERα) to inhibit hormone-dependent proliferation), suppress NF-κB/IL-6/TNF-α-mediated chronic inflammation (e.g., *Frankincense-Myrrh* blocks NF-κB nuclear translocation), restore proliferation-apoptosis balance via PI3K/Akt/MAPK/Bcl-2/Bax pathways (e.g., *Sparganii Rhizoma-Curcumae Rhizoma* induces G0/G1 phase arrest), and enhance antioxidant enzyme (superoxide dismutase [SOD]/glutathione peroxidase [GSH-Px]) activity to alleviate oxidative stress (e.g., *Bupleuri Radix-Angelicae Sinensis Radix* reduces malondialdehyde [MDA] levels). This “multi-component, multi-target, multi-pathway” regulatory pattern addresses the limitations of Western medicine’s single-target intervention, providing a holistic solution to HMG’s complex pathology.

Second, clinical evidence confirms the translational value of these herbal pairs. Clinical studies demonstrate that they alleviate breast pain and reduce lump size with an overall effective rate of 70%–85%, and they exhibit fewer side effects (e.g., no endometrial hyperplasia) compared to conventional Western drugs like tamoxifen. Additionally, this review has established a “Pathogenesis-Syndrome-Herb Pair-Mechanism” precision matching system, classifying herbal pairs by disease stage (early qi stagnation, mid-stage phlegm-blood stasis, late-stage kidney deficiency) and clarifying their correspondence to specific TCM syndromes (e.g., *Cyperi Rhizoma-Curcumae Radix* for liver qi stagnation, *Epimedii Folium-Antler* for Chong-Ren meridian disharmony). This system resolves the long-standing lack of systematic guidance for “syndrome-guided herb pair selection” in clinical practice, laying a foundation for personalized HMG treatment.

Furthermore, this review bridges TCM compatibility theory with modern pharmacology. For each herbal pair, it elaborates on TCM compatibility logic (e.g., *Bupleuri Radix-Angelicae Sinensis Radix* “soothes the liver without consuming liver blood, nourishes blood without stagnating liver qi”) and deciphers the synergistic material basis (e.g., saikosaponins from *Bupleuri Radix* and Z-ligustilide from *Angelicae Sinensis Radix*) and integrated molecular mechanisms. This establishes a direct link between TCM’s “seven emotions compatibility theory” and multi-component synergism, advancing herb pair research from “metabolite enumeration” to “synergistic logic dissection.” The constructed four-tier evidence chain (“network pharmacology prediction → *in vitro* validation → *in vivo* animal models → clinical efficacy”) also enhances the reliability of mechanistic conclusions, addressing the fragmentation of evidence in previous reviews.

Despite these advancements, current research has limitations: complementary regulatory effects of herbal pairs lack quantitative verification (e.g., no Combination Index calculation), clinical studies are mostly small-sampled and short-duration, bioavailability of active metabolites remains unclear, and animal models poorly mimic the chronic cyclic nature of human HMG. These gaps underscore the need for future targeted efforts.

Looking forward, high-quality multi-center, double-blind randomized controlled trials (RCTs) with extended follow-up (1–3 years) are essential to confirm long-term efficacy and safety. Quantitative validation of synergistic mechanisms via *in vitro* cell models and Combination Index assays, coupled with UPLC-Q-orbitrap-MS-based pharmacokinetic studies to clarify the tissue distribution of active metabolites, will further refine herbal pair applications. Additionally, optimizing animal models to incorporate menstrual cycle fluctuations or emotional stress (mimicking TCM “liver qi stagnation”) and exploring integrated TCM-Western regimens (e.g., herbal pairs combined with low-dose tamoxifen) will accelerate clinical translation.

In conclusion, this review provides comprehensive evidence for the efficacy and mechanisms of TCM herbal pairs in treating HMG, validating TCM’s holistic therapeutic philosophy with modern science. These findings not only offer a scientific basis for personalized HMG treatment but also pave the way for the international recognition and application of TCM herbal pairs. Moreover, the potential of herbal pairs to inhibit HMG’s progression to breast cancer (a key risk factor) warrants further exploration, making them promising candidates for both HMG treatment and breast cancer prevention.
